# Expression profile of Epstein-Barr virus and human adenovirus small RNAs in tonsillar B and T lymphocytes

**DOI:** 10.1371/journal.pone.0177275

**Published:** 2017-05-25

**Authors:** Farzaneh Assadian, Wael Kamel, Göran Laurell, Catharina Svensson, Tanel Punga, Göran Akusjärvi

**Affiliations:** 1 Department of Medical Biochemistry and Microbiology, Uppsala Biomedical Center, Uppsala University, Uppsala, Sweden; 2 Department of Surgical Sciences, Otorhinolaryngology and Head and Neck Surgery, Uppsala University, Uppsala, Sweden; University of Nebraska-Lincoln, UNITED STATES

## Abstract

We have used high-throughput small RNA sequencing to characterize viral small RNA expression in purified tonsillar B and T lymphocytes isolated from patients tested positive for Epstein-Barr virus (EBV) or human adenovirus (HAdV) infections, respectively. In the small set of patients analyzed, the expression profile of EBV and HAdV miRNAs could not distinguish between patients diagnosed with tonsillar hypertrophy or chronic/recurrent tonsillitis. The EBV miR-BART expression profile among the patients diagnosed with tonsillar diseases resembles most closely the pattern seen in EBV+ tumors (Latency II/I). The miR-BARTs that appear to be absent in normal EBV infected cells are essentially all detectable in the diseased tonsillar B lymphocytes. In the EBV+ B cells we detected 44 EBV miR-BARTs derived from the proposed BART precursor hairpins whereof five are not annotated in miRBase v21. One previously undetected miRNA, BART16b-5p, originates from the miR-BART16 precursor hairpin as an alternative 5´ miR-BART16 located precisely upstream of the annotated miR-BART16-5p. Further, our analysis revealed an extensive sequence variation among the EBV miRNAs with isomiRs having a constant 5´ end but alternative 3´ ends. A range of small RNAs was also detected from the terminal stem of the EBER RNAs and the 3´ part of v-snoRNA1. During a lytic HAdV infection in established cell lines the terminal stem of the viral non-coding VA RNAs are processed to highly abundant viral miRNAs (mivaRNAs). In contrast, mivaRNA expression in HAdV positive tonsillar T lymphocytes was very low. The small RNA profile further showed that the 5´ mivaRNA from VA RNAI and the 3´ mivaRNA from VA RNAII were as predicted, whereas the 3´ mivaRNA from VA RNAI showed an aberrant processing upstream of the expected Dicer cleavage site.

## Introduction

MicroRNAs (miRNAs) are a large family of ~22-nucleotide (nt) noncoding RNAs expressed in multicellular eukaryotes and also encoded by some viruses [1]. Cellular miRNAs are important components of gene regulatory networks, acting as regulators of diverse cellular processes such as the innate and adaptive immune response, cell differentiation, metabolism, apoptosis, cell proliferation, cancer and maintenance of homeostasis during stress. In humans, more than 2500 mature miRNA species have so far been reported [2].

In the canonical miRNA biogenesis pathway, the miRNA genes are transcribed by RNA polymerase II into a long primary miRNA transcript that is processed by the nuclear Drosha/DGCR8 microprocessor complex, generating a 60–90 nt precursor miRNA (pre-miRNA) hairpin. After being exported to the cytoplasm the pre-miRNA is further processed by the endonuclease Dicer to yield a ~22-nt double-stranded miRNA composed of so-called 5p and 3p strands. One strand of the mature miRNA duplex (the guide strand) is loaded onto one of four Argonaute proteins (Ago) forming the so-called RNA-induced silencing complex (RISC) [3]. Through base pairing, the guide strand directs the RISC complex to the target mRNAs for subsequent post-transcriptional gene silencing [1].

A number of DNA viruses encode their own miRNAs [4]. However, relatively little is known about the function(s) of these viral miRNAs [5]. Like host miRNAs, individual viral miRNAs have numerous potential targets, but only a fraction of these targets has been ascribed a meaningful biological function. Biogenesis of the viral miRNAs is mediated by cellular factors, and so far no evidence of viral proteins regulating/modulating the viral miRNA processing has been described [5].

Most reported viral miRNAs are encoded by the herpesvirus family, in which more than 200 unique mature miRNAs have been characterized [4]. Available data suggest that some of these viral miRNAs serve an important function in the establishment and/or maintenance of long-term latent infections [5, 6].

Epstein-Barr virus (EBV) is a gamma herpesvirus, which infects most individuals in childhood or early adulthood. A childhood infection is usually mild, but if the primary infection occurs during adolescence or later, EBV can cause infectious mononucleosis. After the primary infection, EBV establishes a life-long latent infection, mainly in memory B-cells [7]. Reactivation is usually asymptomatic, but in the absence of competent immune surveillance, B cell malignancies like Hodgkin’s and Burkitt’s lymphoma as well as some solid types of cancer may develop (reviewed in ref. [8]). Latently EBV infected cells circulate to different organs and their presence in the palatine tonsils serve as the source for efficient dissemination of reactivated virus through saliva [9, 10]. In the palatine tonsils, EBV is capable of infecting mainly naive B lymphocytes and lymphoepithelial cells, but also T lymphocytes [11–13].

**Fig 1:**
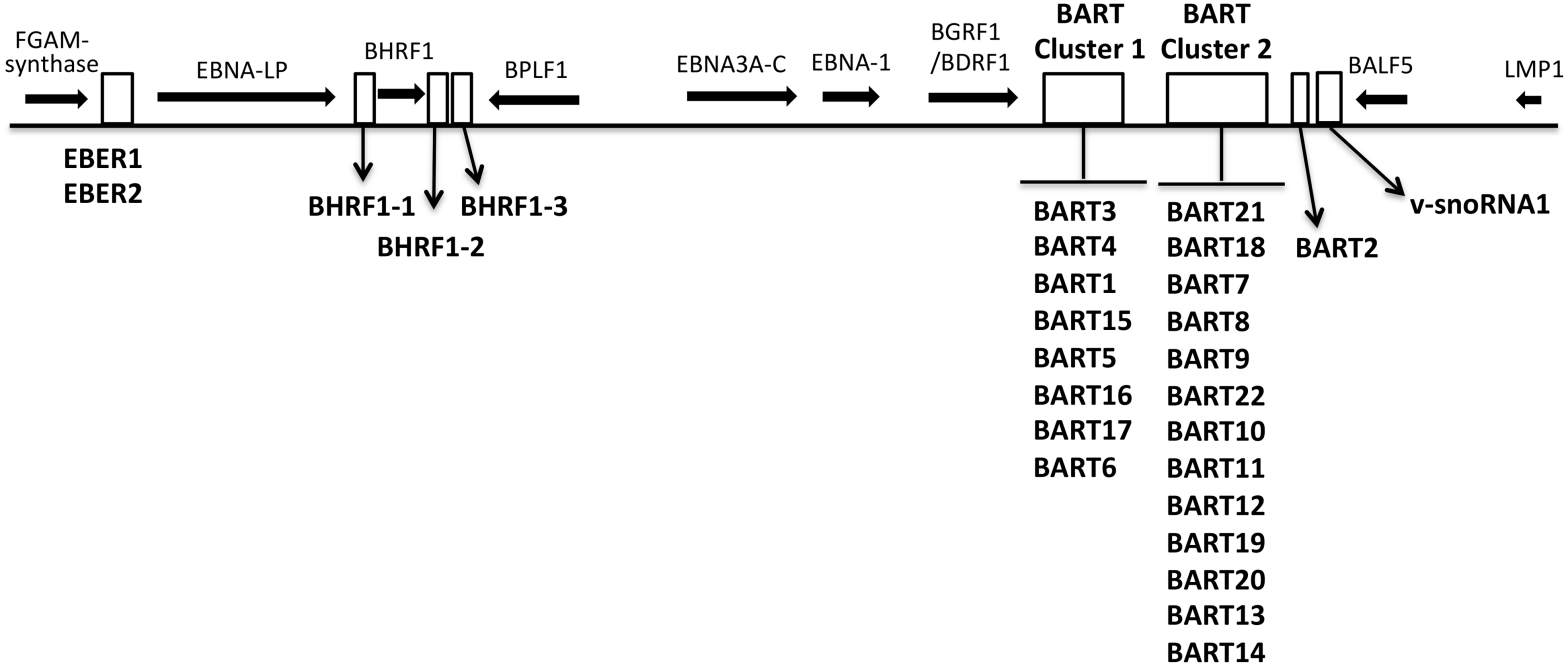
A schematic overview of the genomic location of EBV miRNAs. The BHRF1 and BART miRNA clusters as well as the EBER and v-snoRNA1 transcription units are depicted at their approximate genomic location (white boxes). Black horizontal arrows indicate the relative position of some EBV genes.

EBV encodes 44 characterized mature miRNAs originating from the BHRF1 gene and two clusters within the BART gene (Fig 1), which are differentially expressed during the lytic and latent EBV infection [14–16]. The BHFR1-derived miRNAs are highly expressed in proliferating lymphoblastoid cell lines (Latency III), where also a limited number of miR-BARTs are expressed [15, 17]. In EBV+ tumor cells, in contrast, BHFR1 miRNA expression is drastically reduced and essentially all the miR-BARTs are expressed [15].

The more than 70 human adenovirus (HAdV) types that have been characterized so far are grouped into seven distinct species [18, 19]. As a common human pathogen HAdV is responsible for a variety of clinical diseases such as respiratory tract infections, gastroenteritis and epidemic keratoconjunctivitis [20–23]. HAdVs cause both short-term lytic infections, particularly in epithelial cells, and long-term persistent/latent infections in lymphoid cells. HAdV infection of palatine tonsils and adenoids often results in tonsillar hypertrophy or chronic/recurrent tonsillitis [24–28]. Virus typing experiments have demonstrated that the large majority of patients with tonsillar diseases exhibit HAdV-5 in the T lymphocyte fraction suggesting that tonsillar T lymphocytes might serve as the reservoir for a persistent/latent form of HAdV infection [25, 29].

Both EBV and HAdVs encode for abundant noncoding short RNAs. The EBV genome encodes the EBER RNAs (EBER1 and EBER2) (reviewed in ref. [30]). Similarly, HAdV encodes two virus-associated RNA (VA RNAI and VA RNAII) (reviewed in ref. [31]). The EBER and VA RNAs are short (160–170 nucleotides) highly structured RNAs, which are transcribed by RNA polymerase III. The EBERs and VA RNAs exhibit strikingly similar stem-loop secondary structures [32] and have been ascribed multiple functions [31, 33]. Among these, the EBERs have been shown to bind and inhibit activation of the interferon-inducible double-stranded RNA-dependent protein kinase (PKR) [34, 35], leading to resistance to apoptosis [36–38]. Further, the EBER RNAs have been implicated in modulating host cell gene expression to promote cell proliferation and maintain latency [39]. Thus, EBER RNAs appear to serve complex functions both during a lytic and a latent infection. VA RNAI serves one well-characterized function during a lytic HAdV infection. Notably, it binds PKR and blocks its activation, thereby maintaining the translational capacity of the infected cell (reviewed in ref. [31]). Several studies have shown that the terminal stem of the VA RNAs is processed by Dicer into small viral miRNAs (so-called mivaRNAs) that are incorporated onto active Ago2-containing RISC complexes [40–43]. However, the physiologically relevance of the mivaRNAs for a lytic HAdV-5 infection is not clear [44]. Similarly, the EBERs are processed into small RNAs, although in a Dicer independent manner [45].

One EBV-encoded small nucleolar RNA (v-snoRNA1) has also been detected in EBV-infected cell lines. V-snoRNA1 is 65 nt in length and mapped around 100 bp downstream of the miR-BART2 gene (Fig 1). V-snoRNA1 adopts a secondary structure that resembles pre-miRNA and can be processed into small RNAs by the Dicer enzyme [46, 47].

The aim of this study was to establish the EBV and HAdV small RNA expression profiles in tonsillar B and T lymphocytes. Most of the current knowledge regarding the virus-encoded miRNAs comes from cell culture experiments performed with laboratory virus isolates infecting established cell lines. Such experiments suffer from lack of formal proof in the natural host. In the current project we have used high-throughput small RNA sequencing to characterize EBV and HAdV small RNA expression in B and T lymphocytes recovered after tonsillectomies. In patient tonsillar lymphocytes carrying EBV or HAdV, we detected both previously characterized EBV and HAdV miRNAs, as well as new uncharacterized EBV miRNAs. The expression profile of the EBV miRNAs in the patient samples was reminiscent of that observed in EBV+ tumor cells with a Latency II/I program. Collectively, the data from this study provides a better insight into the nature of the “true” EBV and HAdV viral small noncoding RNA world.

## Materials and methods

### Ethics statement

The study protocol was approved by the Uppsala Ethical Review Board (Dnr. 2013/387/2). Prior to participation in the study, the written informed consent was obtained from the patients or their guardians.

### Clinical specimens

Left and right palatine tonsils were obtained from patients, diagnosed with symptomatic tonsillar hypertrophy or chronic/recurrent tonsillitis and subjected to routine tonsillectomy or tonsillotomy at Uppsala University Hospital, Sweden. The cohort consisted of 55 patients (age 1 to 58) with tonsillar hypertrophy and 56 patients (age 2 to 42) with chronic/recurrent tonsillitis and was previously characterized with respect to HAdV serotype and EBV and HAdV genome content in the purified tonsillar B or T lymphocyte fractions [25, 48]. In the present study we assessed the expression profile of small RNAs in the samples showing the highest EBV (9 samples from 9 patients) and HAdV (11 samples from 11 patients) DNA copy number (Table 1).

**Table 1:** Tonsillar samples used in this study. Right or left tonsils (R or L) were used to purify B or T lymphocytes (B or T).

Sample	Viral DNA copy number/10^6^ cells	Diagnosis	Sex/Age	Virus species
4LT	1.5 × 10^3^	Tonsillar hypertrophy	m/3	HAdV-5
94LT	3.6 × 10^3^	Tonsillar hypertrophy	m/3	HAdV-5
9LT	1.2 × 10^4^	Tonsillar hypertrophy	f/4	HAdV-3
24LT	2.1 × 10^3^	Tonsillar hypertrophy	m/3	HAdV-3
79RT	7 × 10^2^	Tonsillar hypertrophy	f/4	HAdV-3
26RT	2.3 × 10^4^	Tonsillar hypertrophy	m/2	HAdV-2
105LT	1.1 × 10^3^	Tonsillar hypertrophy	m/4	HAdV-2
13RT	1.8 × 10^3^	Chronic tonsillitis	f/12	HAdV-5
66LT	1.9 × 10^3^	Chronic tonsillitis	f/29	HAdV-5
81RT	3.2 × 10^2^	Chronic tonsillitis	f/16	HAdV-5
98LT	6 × 10^2^	Chronic tonsillitis	m/30	HAdV-5
3RB	1.5 × 10^4^	Tonsillar hypertrophy	f/4	EBV
26RB	10^3^	Tonsillar hypertrophy	m/2	EBV
31RB	1.9 × 10^4^	Tonsillar hypertrophy	m/22	EBV
32RB	2.6 × 10^3^	Tonsillar hypertrophy	m/16	EBV
47RB	3.8 × 10^3^	Tonsillar hypertrophy	f/31	EBV
84LB	1.6 × 10^3^	Tonsillar hypertrophy	m/8	EBV
89RB	1.8 × 10^3^	Tonsillar hypertrophy	m/6	EBV
107RB	2.5 × 10^3^	Chronic tonsillitis	f/21	EBV
81LB	1.6 × 10^3^	Chronic tonsillitis	m/16	EBV

### RNA extraction

Total RNA was extracted using the Trizol reagent (Invitrogen, Carlsbad, CA) according to the manufacturer's instructions. The quality of the RNA samples was assessed using an Agilent 2100 Bioanalyzer.

### Small RNA sequencing

RNA samples were treated with tobacco acid pyrophosphatase (TAP enzyme, Epicentre, Wisconsin, USA) to generate 5´–monophosphorylated RNAs. The small RNA libraries were prepared using the Truseq small RNA library preparation kit following the manufacturer’s instructions (Illumina, San Diego, CA). The resulting cDNAs were size selected (17–40 nt) by polyacrylamide gel electrophoresis (6% Novex Tris-Borate-EDTA gel; Invitrogen). RNA sequencing (50-bp single reads) was performed on the Illumina HiSeq2000 platform by the Centre for Genomic Regulation, Barcelona, Spain (www.crg.eu).

### Bioinformatic analysis of small RNA reads

The adaptor sequences were first trimmed from the RNA sequence data. Reads longer than 17 nt in length were mapped to the corresponding EBV (AJ507799.2) or HAdV (HAdV-2: NC_001405.1, HAdV-3: NC_011203.1 and HAdV-5: AC_000008.1) reference sequences. Sequencing reads were also mapped to the human reference genome (GRCh38.p5). Mapping to the reference human or viral genomes was performed using bowtie aligner [49] with no mismatch allowed (EBV) or two mismatches allowed (HAdV). The mapping results were visualized using Integrative Genomics Viewer (IGV) [50]. Classification of mapped reads based on the annotation was done using FeatureCounts [51].

## Results

### Distribution of small RNAs in tonsillar lymphocytes

In a recent report we characterized the prevalence of EBV and HAdV infections in tonsillar B and T lymphocytes isolated from patients diagnosed with tonsillar diseases [25]. Since miRNAs have been implicated in a variety of diseases, we decided to define the viral small RNA profiles in the tonsillar B and T lymphocytes from the patients positive for EBV (EBV+) or HAdV (HAdV+) infections using high-throughput small RNA sequencing. Sequencing reads (~25–50 million per sample) were processed and mapped concurrently to the human and EBV/HAdV genomes. The basic characteristics of the tonsil samples used in this study are summarized in Table 1.

Importantly, the distribution of small RNA reads between different classes of genes in the EBV+ B cell (S1A Fig) and HAdV+ T cell samples (S1B Fig) were similar. For example, 13 to 22% of the reads from EBV+ B cell samples and 17 to 31% of the reads from HAdV+ T cell samples were mapped to cellular miRNA sequences annotated in the miRBase v21 database. To determine if a specific miRNA pattern could be used to distinguish the cell type (B lymphocytes versus T lymphocytes) or diagnosis (tonsillar hypertrophy versus chronic/recurrent tonsillitis), we normalized the cellular miRNA data using the TMM (trimmed mean of M values) method [52] and then analyzed the normalized values using the PCA (principle component analysis) method [53] (S2 Fig). The PCA plot confirmed that tonsillar B and T lymphocytes express discrete miRNA profiles. Three out of the four HAdV+ chronic/recurrent tonsillitis T lymphocyte samples and six out of the seven HAdV+ tonsillar hypertrophy T lymphocyte samples were grouped into the same subcluster. However, this method did not discriminate between the EBV+ tonsillar hypertrophy and chronic/recurrent tonsillitis B lymphocyte samples (S2 Fig).

### Expression profile of EBV-encoded miRNAs

**Fig 2:**
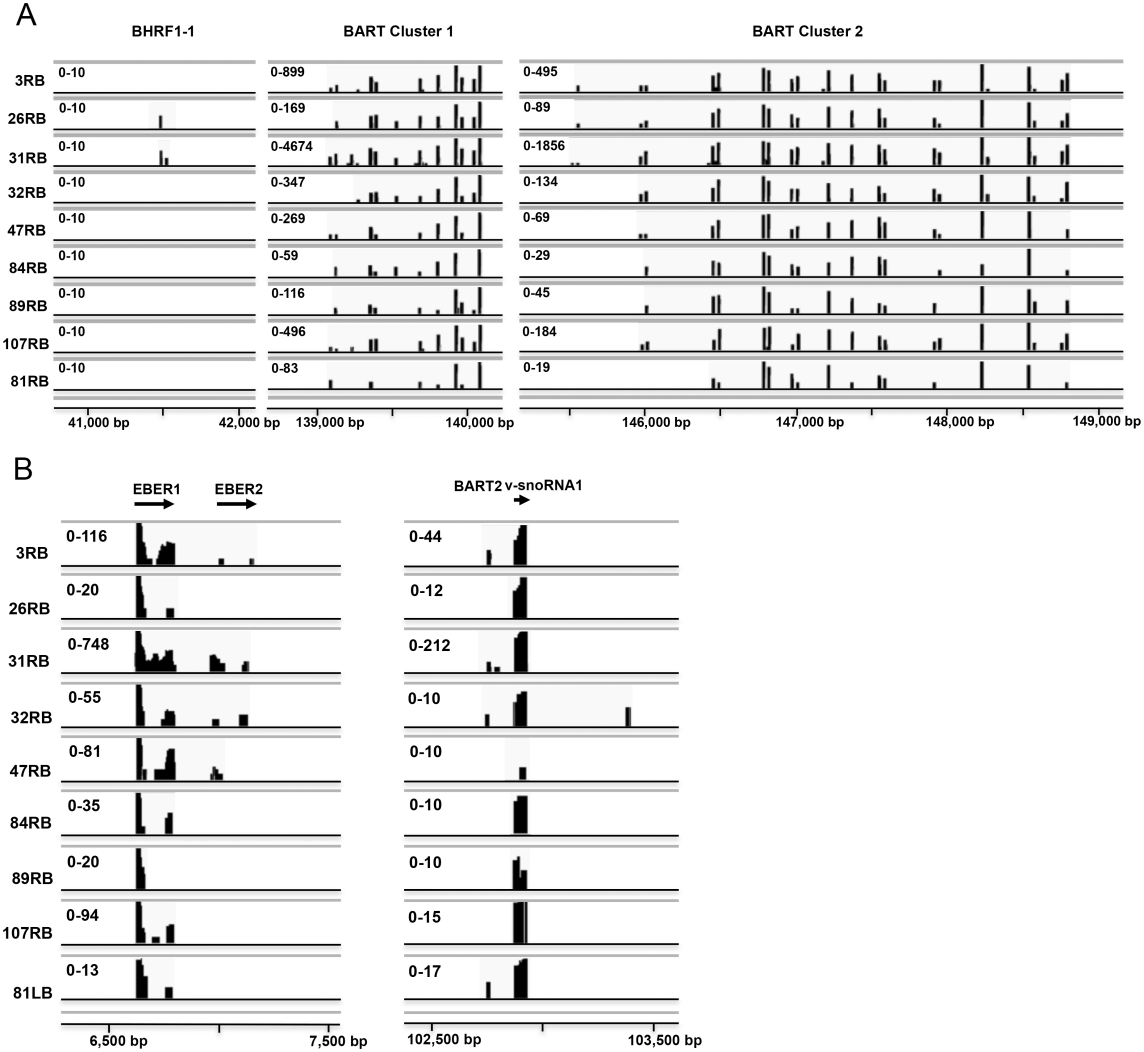
Genomic view of the sequencing reads mapped to the EBV genome. The alignment data are visualized in Integrative Genomics Viewer (IGV). The coverage track shows the position of the EBV BHRF-1-1 and BART miRNAs (A) and EBER1, EBER2 or v-snoRNA1 derived small RNA reads (B). The range of the number of sequencing reads is shown for each patient sample. The abbreviation of patient samples was as follow: first patient number (Table 1) followed by right (R) or left (L) tonsil followed by origin of tonsillar cells (B or T cells).

To profile the expression levels of EBV small RNAs in the purified B lymphocytes, the sequencing reads were aligned to the viral genome. The mapping results were visualized in graphs showing the genomic location of the BHRF1 and BART cluster 1 and 2 (Fig 2A) and the EBER, BART2 and v-snoRNA1 small RNAs (Fig 2B). Approximately 0.14% of the non-human-mapped reads were aligned to the EBV pre-miRNA sequences present in the miRBase v21.

EBV expresses 44 annotated mature miRNAs (miRBase v21) all derived from the 25 proposed precursor hairpins located in the BHRF1 and BART clusters. In the tonsillar B cell samples, we detected 36 of the annotated EBV miRNAs and some previously uncharacterized EBV miRNAs (see below). Interestingly, only two (26RB, 31RB) out of nine samples expressed a miRNA from the Latency III specific BHRF1 cluster (miR-BHRF1-1, Fig 2A and S1 Table) [54].

**Fig 3:**
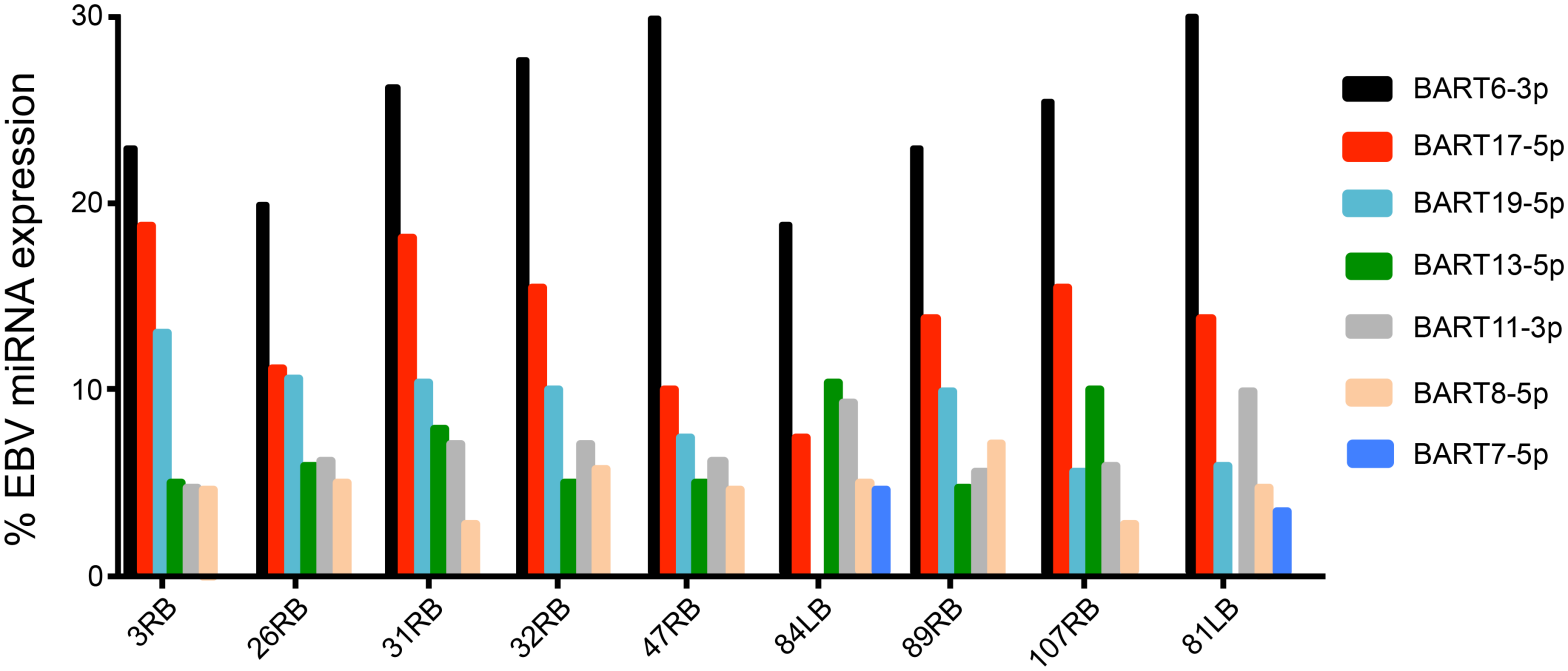
The relative expression of the six most abundant EBV miRNAs in B lymphocytes from individual patients. The relative expression of these miRNAs is shown as percentage of total EBV miRNA reads in the individual patients.

The expression profile of BART cluster 1 and cluster 2 miRNAs in the tonsillar B lymphocytes was very similar between the different patients samples (Fig 2A and Table 2), although the expression level of individual miRNAs differed between patient samples (Fig 2A, see below). Strikingly, miR-BART6-3p and miR-BART17-5p were the most abundant viral miRNAs in all samples, except patient 84LB, where they together accounted for 30% to more than 40% of the total EBV miRNA pool (Fig 3 and S1 Table).

**Table 2:** The number and percentage of the EBV miRNAs corresponding to the miRNA genomic regions are shown in individual samples.

		Number and percentage of the EBV miRNAs								
		3RB	26RB	31RB	32RB	47RB	84LB	89RB	107RB	81LB
GenomicRegion	Total miRNAs present in the region									
BHRF1	5	0 (0%)	1 (20%)	2 (40%)	0 (0%)	0 (0%)	0 (0%)	0 (0%)	0 (0%)	0 (0%)
BART Cluster1	17	14 (82%)	13 (76%)	15 (88%)	11 (65%)	10 (59%)	8 (47%)	10 (59%)	10 (59%)	8 (47%)
BART Cluster2	28	24 (86%)	23 (82%)	26 (93%)	22 (79%)	21 (75%)	17 (61%)	19 (68%)	22 (79%)	17 (61%)
Total	50	38 (76%)	37 (74%)	43 (86%)	33 (66%)	31 (62%)	25 (50%)	29 (58%)	32 (64%)	25 (50%)

### Unusual mature EBV BART miRNAs in tonsillar B lymphocytes

**Fig 4:**
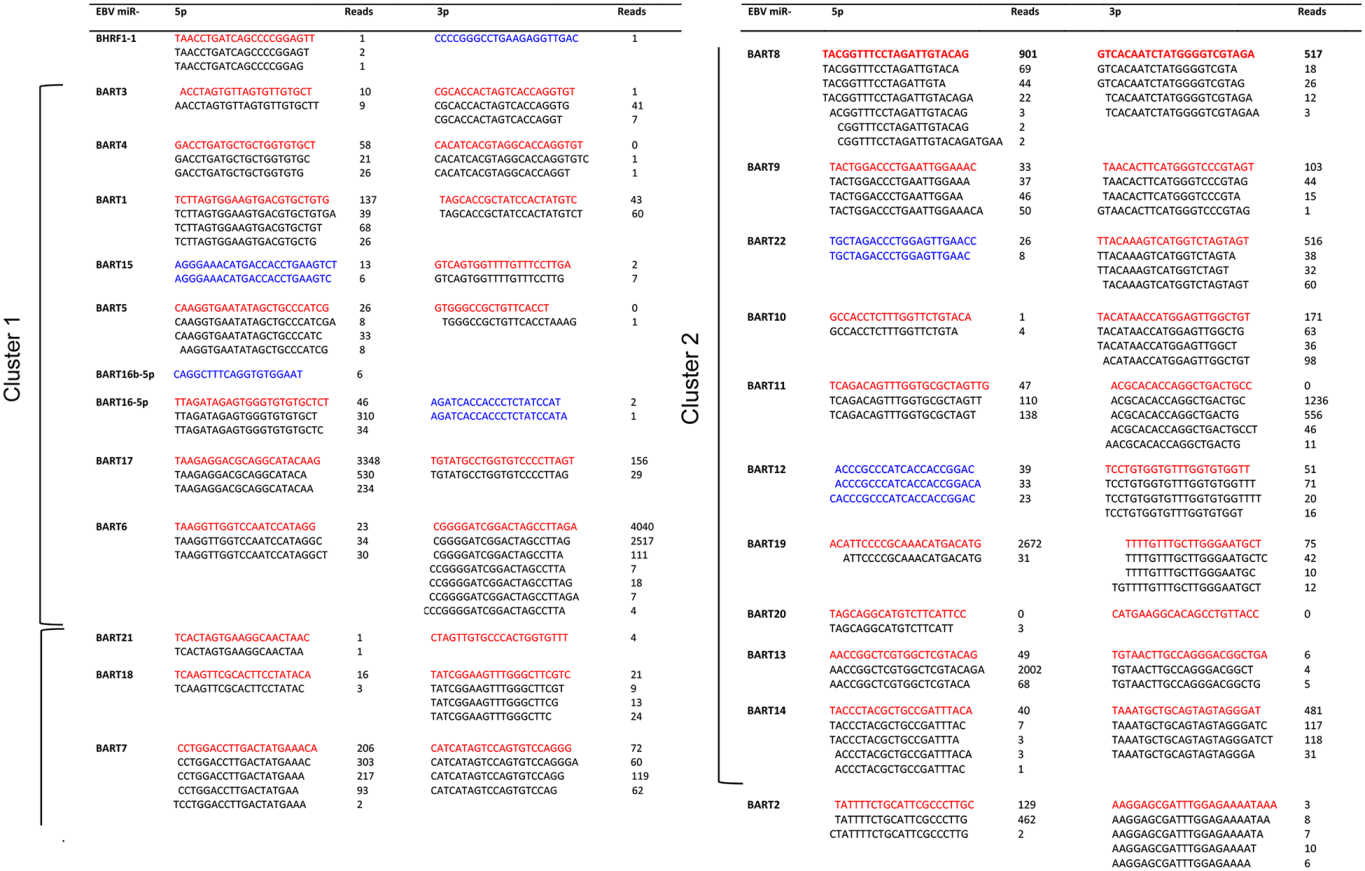
Small RNA expression profile of EBV miRNAs and isomiRs in patient B lymphocytes. The miRBase v21 reference sequence for each miRNA is shown in red and isomiRs of these miRNAs in black. Sequences not annotated in mirBase v21 are shown in blue. The data is shown as the collective number of reads from all nine EBV positive patient samples (Table 1) subjected to small RNA sequencing. The novel BART16b-5p miRNA originates from a position upstream of the characterized miR-BART16-5p (S3 Fig).

Six of the mature EBV miRNAs detected in the tonsillar B lymphocytes have not been annotated in the miRBase v21 database. Five of them appear to be derived from proposed precursor hairpins where only one mature miRNA strand has been annotated in miRBase v21 (BHRF1-1, BART15, BART16, BART22, BART12, Fig 4). Although not reported in the miRBase v21, four of the miRNAs (miR-BART15-5p, miR-BART22-5p and miR-BART12-5p and miR-BART16-3p) have been previously detected in EBV+ cells [55, 56]. The miR-BHRF1-1 miRNAs accumulated at very low levels and the novel miR-BHRF1-1-3p was only detected as one read in one of the patient samples (31RB). The new EBV miRNA (named by us as miR-BART16b-5p) originates from the miR-BART16 precursor hairpin as an alternative 5´ BART16 miRNA, which is juxtaposed precisely upstream of the mature annotated miR-BART16-5p sequence (Fig 4 and S3 Fig). Essentially all identified EBV miRNAs accumulate as high or low abundant isoforms (so called isomiRs [57]) containing alternative 3´ ends and in some cases novel 5´ ends compared to the miRBase v21 annotated sequences. Seventeen of the detected miRNAs displayed a low abundance of 5´ heterogeneous variants, while essentially all of the miRNAs showed a large proportion of 3´ heterogeneous miRNAs (Fig 4).

### Accumulation of EBER- and v-snoRNA1-derived small RNAs in tonsillar B lymphocytes

In addition to the BHRF1 and BART clusters encoded miRNAs, the EBERs and v-snoRNA1 produced small RNAs in the tonsillar B lymphocytes, respectively accounting for approximately 5% and 2% of the total EBV-derived small RNA pool (S2 Table).

**Fig 5:**
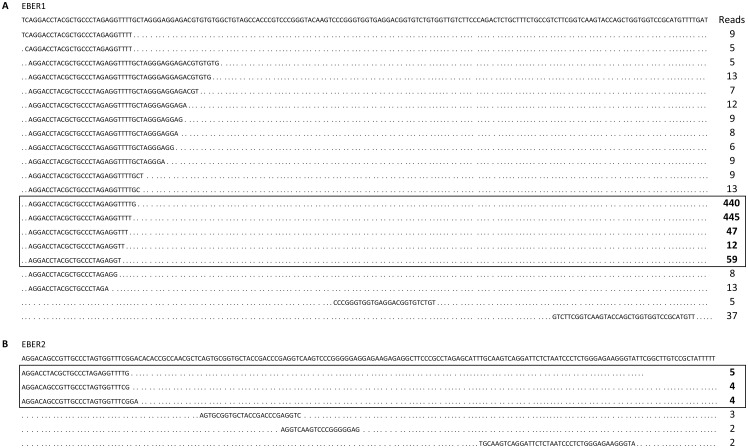
Coverage of small RNA reads originating from EBER RNA1 (A) and EBER RNA2 (B). The top line in both panels shows the full-length sequence of the respective EBER RNA. The nucleotide sequence and read count for each small RNA is indicated. The most highly expressed small RNA fraction is shown boxed in both panels. Only sequences with a read count ≥ 5 (EBER1) or ≥ 2 (EBER2) are displayed.

The majority of the EBER-derived small RNA reads mapped to the 5´ end of both EBER1 and EBER2 (Fig 5 and S4 Fig). In agreement with the differential EBER1 and EBER2 expression [30], most of the EBER-derived small RNAs originated from EBER1. As shown in Fig 5A, a heterogeneous range of small RNAs originating from the EBER1 5´ terminus were detected. Further, the coverage of the EBER1 5´ terminus was dominated by small RNA reads with a length of 23–27 nucleotides. The EBER1 3´ terminus generated much less small RNAs exhibiting heterogeneity at both the 5´ and 3´ ends (less than five total reads each and therefore not displayed in Fig 5A). In some individual patient samples, like 3RB and 31RB and to lesser extent 47RB, reads were also detected from the EBER1 apical stem (S4 Fig).

Although detected at a substantially lower abundance compared to the EBER1 small RNAs, a similar trend was seen in the case of the EBER2-derived small RNAs (Fig 5B). The 5´ small RNAs varied between 27–29 nucleotides in length, with scattered reads from the central region and sequences near the 3´terminus of EBER2.

**Fig 6:**
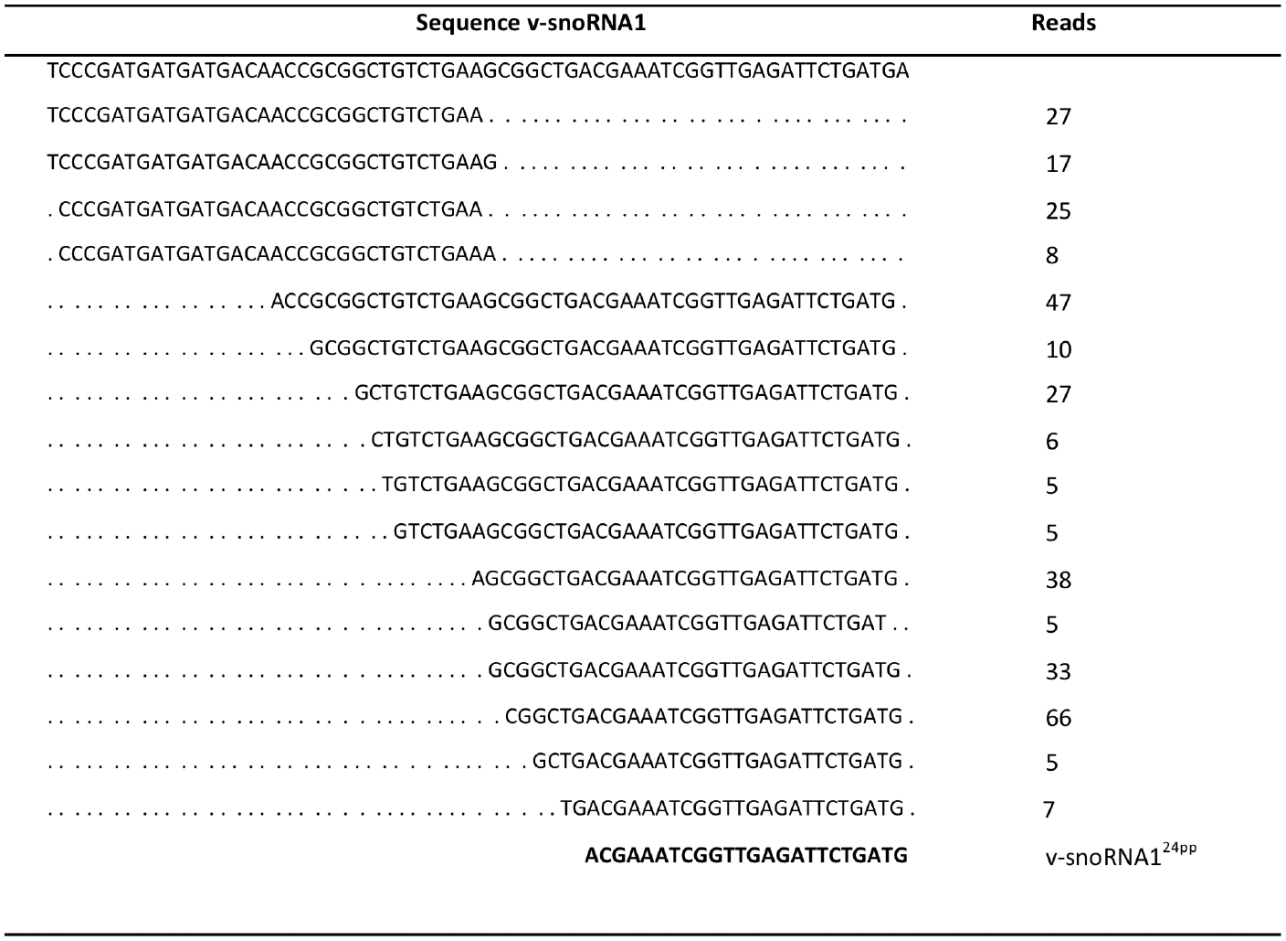
Coverage of small RNA reads originating from v-snoRNA1. The top line depicts the sequence of full-length v-snoRNA1. The data is shown as the collective number of reads from all nine EBV positive patient samples (Table 1) subjected to small RNA sequencing. The sequence of the previously characterized v-snoRNA1^24pp^ [46] is shown in bold at the bottom.

It has been proposed that v-snoRNA1 might serve as a miRNA-like precursor, which is processed by Dicer into small RNA species of different sizes [46, 47]. Indeed, our small RNA sequencing data demonstrated that a large collection of small RNA reads originating from the v-snoRNA1 5´ and 3´ termini were detected in tonsillar B lymphocytes (Fig 6). The large majority of these reads mapped to the 3´ terminus of the v-snoRNA1 gene with varied length between 26–47 nucleotides (Fig 6).

### HAdV mivaRNA expression in tonsillar T lymphocytes

Several studies have utilized high throughput sequencing to characterize viral small RNAs that are produced during HAdV-5 lytic [42, 43, 58, 59] and persistent infections [60]. Collectively, these studies have suggested that the VA RNA genes are the major source for small RNA production during HAdV infections in established cell lines.

**Fig 7:**
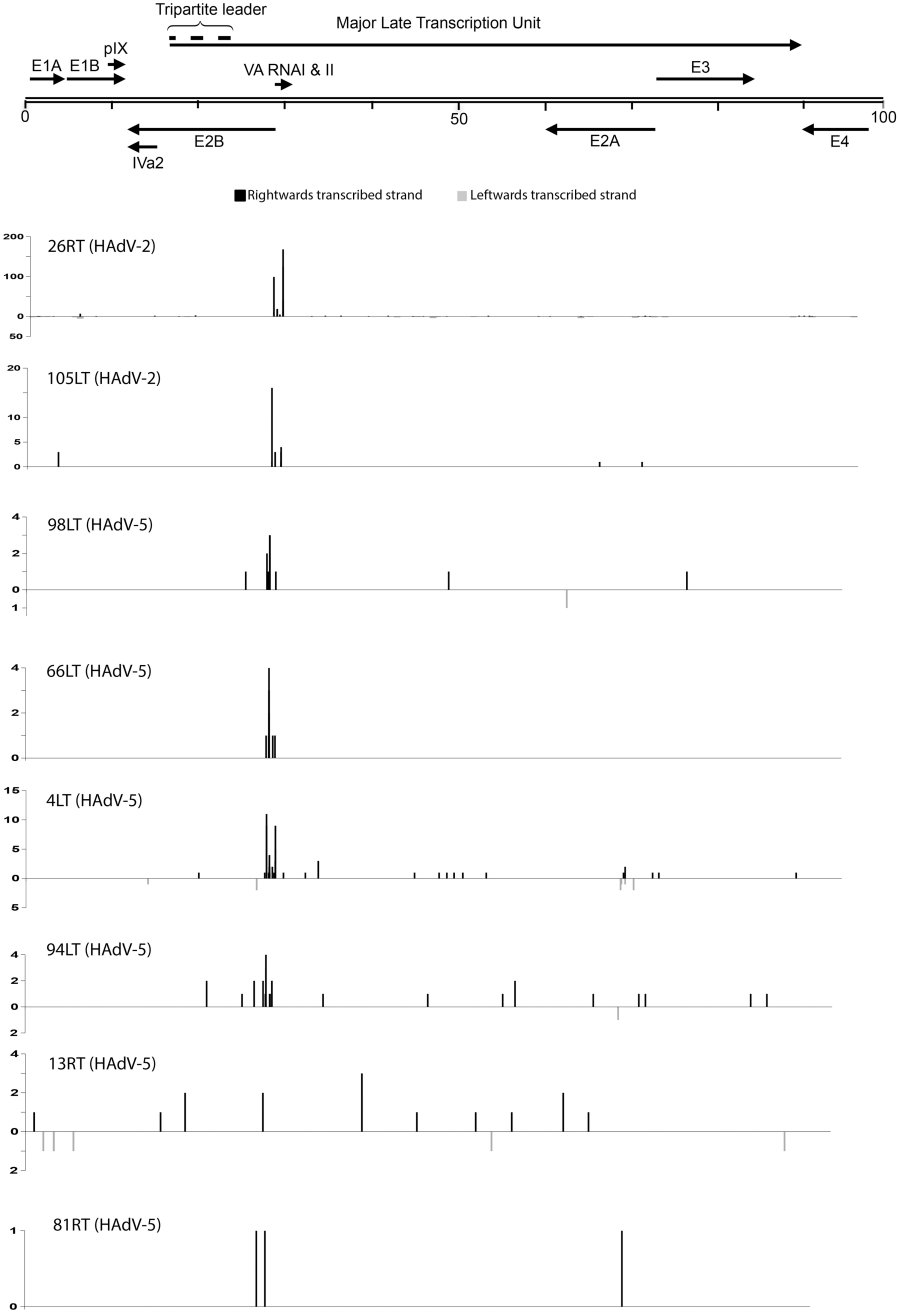
Distribution of small RNAs reads from the HAdV genome in patient tonsillar lymphocytes. A schematic drawing showing the position of HAdV transcription units is shown at the top. Reads derived from the rightwards-transcribed strand is shown with black boxes and reads derived from the leftward-transcribed strand is shown as grey boxes. The number of reads is shown on the y-axis. The abbreviation of patient samples was as follow: first patient number (Table 1) followed by right (R) or left (L) tonsil followed by the origin of tonsillar cells (T or B lymphocytes). In the patient samples diagnosed with a HAdV-3 infection the virus-specific small RNA accumulation was at a background level (data not shown).

Here we investigated the small RNAs accumulating in HAdV+ tonsillar T lymphocytes in patients tested positive for HAdV-2, HAdV-3 and HAdV-5 DNA (Table 1). In comparison to the EBV samples the number of HAdV specific small RNA reads were miniscule (approximately 0.002% of non-human-mapped reads). We therefore allowed for two mismatches during the mapping against the HAdV reference genomes. In patient samples diagnosed with a HAdV-3 infection the virus-specific small RNA accumulation was at a background level with a single read corresponding to the apical stem of the VA RNAI gene. In the HAdV-2 and HAdV-5 positive patient samples the general trend was that the majority of viral RNA reads mapped to the VA RNA region (Fig 7). Further, in the T lymphocytes tested positive for HAdV-2 infection, the number of VA RNA-derived reads was much higher compared to the HAdV-5 containing samples.

**Fig 8:**
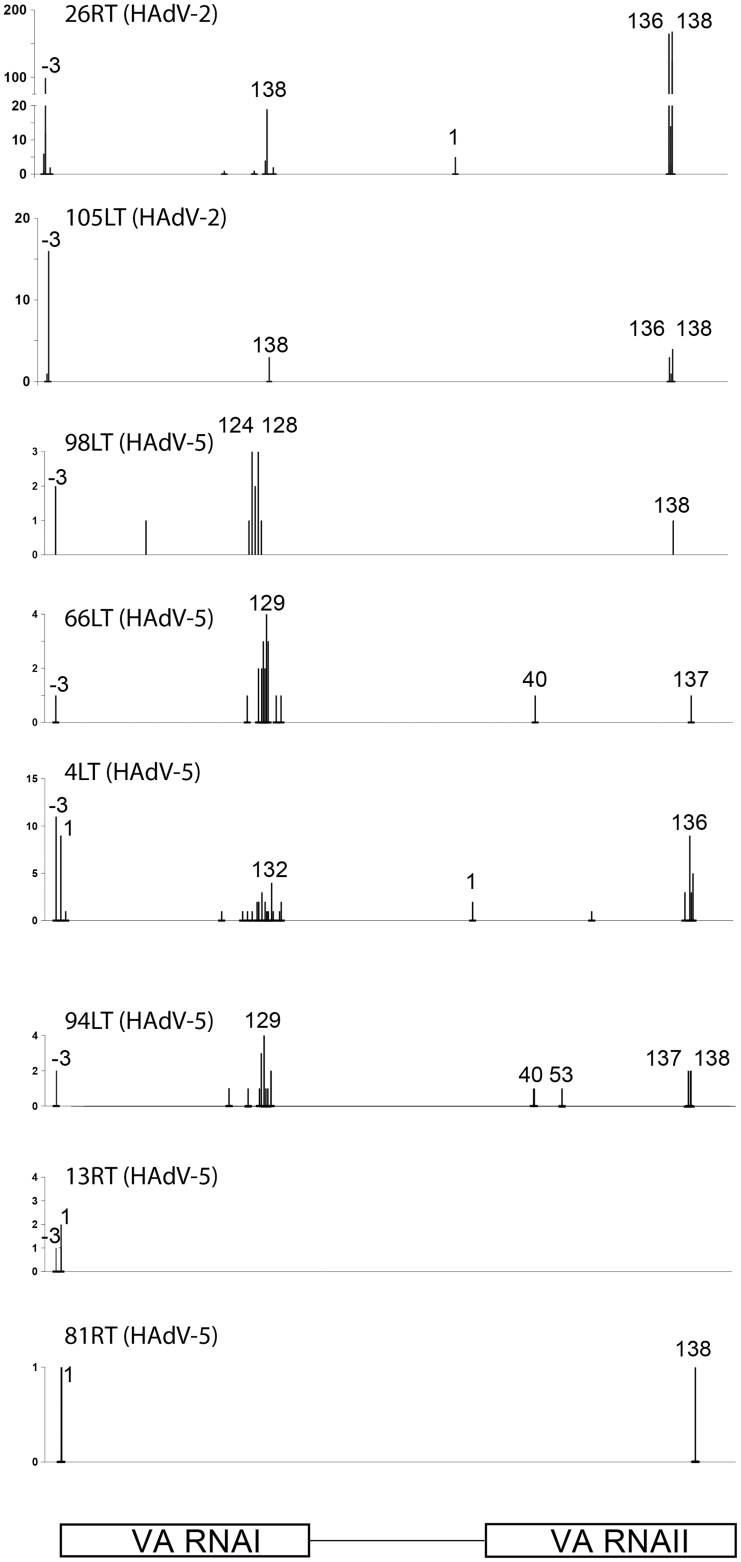
Distribution of small RNA reads from the VA RNAI and VA RNAII genes in patient tonsillar lymphocytes. The nucleotide position of the VA RNAI and VA RNAII small RNA 5´ends are shown in the panels with the number of reads depicted on the y-axis. For a detailed explanation of the nomenclature see Kamel et al. [43]. In the patient samples diagnosed with a HAdV-3 infection only a single VA RNA derived small RNA read was detected (not shown).

Alignment of the HAdV small RNA reads to the respective HAdV genomes showed that between 12 and 99% of the viral small RNA reads in individual patient samples mapped to the VA RNA genes. For both VA RNAI and VA RNAII the large majority of reads mapped to the terminal stem region (Fig 8), a result compatible with the previous conclusion that the mivaRNAs are Dicer cleavage products [43]. For both VA RNAI and VA RNAII the 3´ mivaRNA showed a much higher coverage than the 5´ mivaRNA. In fact, no reads corresponding to the 5´ mivaRNAII could be detected in any of the patient samples (Fig 8). In contrast, the 3´ strand of VA RNAII (3´ mivaRNAII-138) was, by far, the most abundant small RNA detected. This is identical to the major 3´ mivaRNAII species also detected in previous cell culture experiments [42, 43, 58, 59]. In contrast, the 3´ mivaRNAI, which is the most abundant mivaRNA expressed in a lytic infection [43], showed heterogeneous 5´ ends primarily located 10 nucleotides upstream of the characterized Dicer cleavage site at nucleotide 138. In the HAdV-2 samples the mivaRNAI-138 read was by far the most abundant 3´ mivaRNAI (Fig 8), suggesting a potential difference in processing of HAdV-2 and HAdV-5 VA RNAI in tonsillar T lymphocytes. The VA RNAI gene has two transcriptional start sites, which produce a major transcript (75%, VA RNAI(G)) and a 3 nucleotide longer minor transcript (25%, VA RNAI(A)) [61, 62]. Interestingly, in the majority of patient samples the VA RNAI 5´ end coincided with the minor VA RNAI(A) transcriptional start site (Fig 8).

## Discussion

Here we report the expression profiles of EBV- and HAdV-encoded small RNAs in B and T lymphocytes purified from the patient palatine tonsils.

We detected 36 of the 44 previously miRBase v21 annotated mature miRNAs located in the EBV BHRF and BART clusters (Fig 4). We also detected four BART (miR-BART15-5p, miR-BART16-3p, miR-BART22-5p, miR-BART12-5p) and one BHRF miRNA (miR-BHRF1-1-3p) not annotated in miRBase v21, but derived from the proposed precursor hairpins where only one mature miRNA strand has previously been annotated (Fig 4). In line with our data, a recent study has shown that miR-BART15-5p, miR-BART16-3p, miR-BART22-5p and miR-BART12-5p are detectable at very low quantities in EBV+ cancer cell lines [56]. However, since only miR-BART16-3p showed miRNA-like activity in reporter assays, the functional role of these new small RNAs remains to be solved. In addition, the novel miR-BART16b-5p miRNA appears to be processed from the miR-BART16 hairpin (S3 Fig) and positioned immediately upstream of the miRBase annotated miR-BART-16-5p. Hypothetically, the cleavage event generating the 5´ end of miR-BART16-5p also generates the 3´ end of miR-BART16b-5p.

A 5´ and 3´ miRNA heterogeneity has previously been reported in several studies [55–57, 63–66]. We also observe a high degree of heterogeneity, particularly at the 3´ end of the EBV isomiRs (Fig 4). Many of these variants might result from nibbling of nucleotides from the mature EBV miRNAs in cells. However, it appears less likely that the isomiRs are caused by RNA degradation during sample preparation since spike in RNA experiments suggests that shortening of RNAs preferentially occurs at the 5´ end of the small RNA [67]. Since we also detect a small number of reads with longer 5´ or 3´ tails it appears likely that we are detecting non-canonical Dicer processing products [68]. In four cases (miR-BART4-3p, miR-BART5-3p, miR-BART11-3p and miR-BART20-5p), we did not detect the annotated BART miRNA, but instead isomiRs of these annotated EBV miRNA. If we also count these isomiRs as miRNAs the total number of EBV BART miRNAs detected is raised from 35 to a total of 39 EBV BART miRNAs. It should be noted that in all cases, except one, where we detect an isomiR instead of the annotated mature miRNA, the read count is extremely low (miR-BART4-3p, miR-BART5-3p and miR-BART20-5p; Fig 4). The exception is the isomiR forms of miR-BART11-3p, which was the fifth most abundant EBV miRNA in our read count (Fig 4). Although the potential target mRNAs will change for isomiRs with alternative 5´ ends we note, with the possible exception of miR-BART3-5p (Fig 4), that all isomiRs with alternative 5´ ends have minute read counts compared to the corresponding mature miRNA.

Asymptomatic reactivation of EBV from a latent into a lytic infection is believed to occur regularly, the predominant state is the latency phase 0 or I. Since miR-BHRF1-1 overexpression can potentiate induction of an EBV lytic infection [69], the observation that we did not detect BHRF1 miRNA expression in 7 out of 9 patient (Fig 2A) is consistent with the suggestion that most patients harbor a latent EBV infection with undetectable reactivation of a lytic phase. This is further supported by the very low accumulation of miR-BHFR1-1 and its isomiRs in the two patients samples (26RB and 31RB). Also, a single read of the miR-BHRF1-1 variant (miR-BHFR1-1-3p) that we detect in patient sample 31RB is novel and has not previously been described (Fig 4). The expression profile of cluster 1 and 2 miR-BARTs in the tonsillar B lymphocytes was similar between different patients samples (Fig 2A and S1 Table) and most of the annotated miR-BARTs were detected in all patients. In fact, 8 to 15 out of the 17 miR-BART cluster 1, and 17 to 26 out of the 28 miR-BART cluster 2 miRNAs were observed in the individual patient samples. Furthermore, our profiling reveals that miR-BART6-3p and miR-BART17-5p were the first and second most abundant EBV miRNAs in the patient derived tonsillar B cell population (Fig 3).

Interestingly, the miR-BART expression profile we detect in tonsillar B lymphocytes is reminiscent of the pattern observed in various EBV+ tumor cell lines derived from nasopharyngeal cancer, gastric carcinoma and Hodgkin´s disease (all Latency II) or Burkit’s lymphoma (Latency I) patients [15]. The EBV miRNA expression profile has also been characterized in normal infected cells, like tonsillar germinal center B cells (GCB, Latency II) and memory B cells (MemB, Latency I/0) [15]. In these cells BHFR1-derived miRNAs and a large fraction of the cluster 1 and cluster 2 BART miRNA were absent. Strikingly, in EBV+ tumor cells BHFR1 miRNA expression was drastically reduced and essentially all cluster 1 and 2 BART miRNAs were expressed. Based on our profiling experiments, at least four EBV miRNAs (miR-BART7-5p, miR-BART10-3p, miR-BART13-5p, miR-BART14-5p), which were absent in the GCB and MemB cells, were detected in patient-derived tonsillar B cells. Since these EBV miRNAs also are expressed in the aforementioned EBV+ tumor cells [15], it is possible that their expression in our patient derived tonsillar B lymphocytes correlates with a specific pathogenic EBV infection state, such as a Latency II/I program. Interestingly, miR-BART11-3p was not detected in GCB or MemB cells but was highly expressed in EBV+ tumor cells [15]. In our study we did not detect a single read corresponding to the canonical miR-BART11-3p. Instead we detected high numbers of miR-BART11-3p isomiRs with one or two nucleotides missing at their 3´end (Fig 4).

There is a complex interplay between EBV miRNAs and viral or cellular target transcripts. The viral targets of some of the EBV miRNAs are easy to determine because they are transcribed as antisense sequences to the viral genes [6, 70–72]. The exact function of the majority of viral miRNAs has yet to be fully understood, although most of the known miR-BART targets are involved in extending infected cell viability, enhancing proliferation during latency establishment or evasion of the host immune response [5, 6].

Since all the samples in the current study exhibit the same set of highly abundant EBV miRNAs, we assume that these miRNAs play a crucial role in EBV pathogenesis possibly by contributing to the establishment and/or maintenance of long-term latent infections or malignant cell transformation. For example, the most highly expressed miR-BART6-3p (Fig 3) has been shown to play an important role in the pathogenesis of Burkitt’s lymphoma by reducing IL-6 receptor and phosphatase and tensin homolog (PTEN) expression. Both proteins control vital cellular functions such as cell proliferation, apoptosis, and immune surveillance. Impairment of these key cellular pathways might result in immune evasion and malignant transformation of the infected cells [73, 74].

Also, BART17-5p, which was the second most highly expressed EBV miRNA in our patient samples (Fig 3), together with miR-BART1-5p and miR-BART16 inhibits the expression of LMP1 gene in nasopharyngeal carcinoma cells [71]. A reduced expression of the LMP1 gene is vital for cell survival [47, 71, 75, 76], since LMP1 overexpression induces NF-κB-dependent apoptosis [77, 78]. In addition, miR-BART17-5p, together with miR-BART19-3p and miR-BART7 down regulates the expression of tumor suppressor gene adenomatous polyposis coli (APC), which is a well-known Wnt antagonist [79]. Therefore, these miR-BARTs can activate the Wnt signaling pathway, which in turn induce the proliferation of EBV-infected epithelial cells [79, 80]. MiR-BART2 targets the 3´ UTR of the viral DNA polymerase gene (BALF5). Since BALF5 is required for the lytic phase of an EBV infection, miR-BART2 could control the latent-lytic switch by limiting BALF5 expression [70]. MICB (major histocompatibility complex class I-related chain B) is another target for miR-BART2. MICB is a stress-induced ligand for NK cells and CD8+ T cytotoxic cells, so down regulation of this protein results in less cell dependent cytotoxicity of the EBV-infected cells [81, 82]. Inhibition of apoptosis might be achieved by EBV miR-BART5 targeting the cellular pro-apoptotic PUMA gene [83]. EBV miR-BHRF1-3 has been shown to reduce CXC-chemokine ligand 11 expression, which might help an infected cell to hide from T cell recognition [84].

In addition to the conventional miRNAs, EBV encodes the EBER RNAs and v-snoRNA1, which also produce miRNA-like small RNAs. The EBER-derived small RNAs are heterogeneous in size and generally originating from the 5´ side of the terminal stem (Fig 5). While these EBER-derived small RNA fragments have been detected by northern blot and deep sequencing methods in other studies, no strong experimental evidence supports that they are bona fide miRNAs [45, 47]. It is possible that they might be unspecific breakdown products of the highly abundant EBERs [47, 64].

It has been suggested that v-snoRNA1 might act as a miRNA precursor [46] and become processed by Dicer into miRNA-like molecules [47]. We identified a large number of small RNA reads, which mapped to the 5´ and particularly to the 3´ termini of the v-snoRNA1 gene (Fig 6). Essentially all of the RNA reads have the correct 3´ nucleotide position (98%) but differ by having additional nucleotides at the 5´ end of the characterized v-snoRNA1^24pp^. The v-snoRNA1 gene is transcribed antisense to the 3´ UTR of the viral DNA polymerase gene, BALF5, and therefore could theoretically play a role in the viral life cycle by down regulating BALF5 expression [46]. In contrast to our result, in cultured EBV-infected B lymphocytes, Hutzinger *et al*. [46] identified only a single 24 nt small RNA, designated as v-snoRNA1^24pp^ from the 3´ terminus of v-snoRNA1 which was not detectable in our small RNA sequence data (Fig 6).

Both sides of the terminal stem of the VA RNAs from all tested HAdV types produce miRNA-like small RNAs (the mivaRNAs) during virus growth in established cell lines [42, 43, 58, 59]. These mivaRNAs accumulate to large amounts at the late stage of a lytic HAdV-5 infection and constitutes more than 99% of all small RNAs derived from the HAdV-5 genome. The mivaRNA expression has also been characterized in HAdV-5-infected B and T cell lines that appear to undergo a persistent infection [60]. Here we will compare the mivaRNA production in established cell lines with the patient-derived tonsillar T lymphocytes.

The accumulation of mivaRNAs in the tonsillar T lymphocytes was surprisingly low (0.002%) compared to the EBV-specific small RNAs in B lymphocytes that accounted for approximately 0.14% of non-human-mapped reads. This was surprising considering that the DNA copy number was similar between the HAdV+ and EBV+ patient samples (Table 1). We do not know whether this low expression profile of mivaRNAs in tonsillar T lymphocytes is due to experimental artifacts or reflects the fact that the VA RNAs are not processed into mivaRNAs during HAdV infection in the tonsillar T lymphcytes. Further, we do not detect any distinct difference in the mivaRNA profiles (Fig 8) between patients diagnosed with tonsillar hypertrophy or chronic/recurrent tonsillitis (Table 1, S2 Fig).

In HAdV-5-infected established cell lines (HEK293, IMR90, A549) the major small RNA accumulating is the 3´ mivaRNAI-138 derived by processing of VA RNAI. The mivaRNAI-138 species was also the major mivaRNAI detected in the HAdV-2 infected T cells. Surprisingly, this species was undetectable in the HAdV-5 patient samples (Fig 8). Instead, HAdV-5 samples displayed an array of differently processed 3´ mivaRNAI with a peak indicating a processing site at nucleotide 128 (Fig 8). This is the same site as has previously been observed in persistently infected lymphoid cell lines [60]. These data may suggest that despite the sequence similarity between HAdV-2 and HAdV-5 VA RNAI [85] there might be other factors governing its processing into small RNA. The major mivaRNA detectable in patient T lymphocytes was mivaRNAII-138, which is derived from the 3´ strand of VA RNAII (Fig 8). Interestingly, the same small RNA is the major 3´ mivaRNAII expressed during a lytic HAdV-5 infection. The expression profile of the mivaRNAs in patient T cells was very similar to the mivaRNAI and mivaRNAII expression observed in persistently infected lymphoid cell lines [60].

A surprising result was that the large majority of the 5´ mivaRNAs derived from VA RNAI had a 5´ end coinciding with the minor VA RNAI(A) transcriptional start site (Fig 8), which accounts for only 25% of VA RNAI species expressed during a lytic infection [61]. This might be significant since we have previously shown that the mivaRNA derived from the VA RNAI(A) transcriptional start site generates active RISC complexes capable of inducing RNAi [62]. The mivaRNA generated from the VA RNAI(G) transcriptional start site was more efficiently assembled into RISC but generated unstable RISC complexes with a low cleavage activity. The extremely low level of mivaRNA expression in the patient-derived tonsillar T lymphocytes might suggest that the mivaRNAs are not key regulators of establishment or maintenance of persistent HAdV infections in the palatine tonsils.

## Supporting information

S1 Fig Genotyping of the sequencing reads aligned to human genome. The pie charts display the distribution of the mapped reads from EBV+ (A) and HAdV+ (B) samples.
(PDF)

S2 Fig Principle component analysis (PCA) of cellular miRNA expression in the tonsillar B and T lymphocytes. The PCA analysis was performed on the data set normalized based on the TMM method. For the T cell patient samples the diagnosis (tonsillar hypertrophy versus chronic/recurrent tonsillitis) are shown circled.
(PDF)

S3 Fig The proposed EBV miR-BART16 precursor RNA. The annotated BART16-5p miRNA is shown in red whereas the new BART16-3p and BART16b-5p are shown in blue.
(PDF)

S4 Fig Coverage of EBER derived small RNA reads in the EBV+ B lymphocytes from the different patients. The boxes indicate the relative distribution of reads mapped to 5´or 3´regions of EBER1 and EBER2.
(PDF)

S1 Table The expression level of the miRNAs in the individual patient samples is shown as the percentage of each miRNA relative to the total miRNA content. The asterisk denotes new miRNAs not annotated in miRBase v21. The highest-expressed miRNAs in each sample are shown in bold.
(PDF)

S2 Table Expression level of the EBER- and v-snoRNA1-derived small RNAs and the highest expressed BART miRNAs in the EBV-infected B lymphocyte patient samples. The expression level of the small RNAs/miRNAs in individual patients is shown as the percentage that each RNA contributes to the total EBV specific small RNA pool.
(PDF)

## References

[1] Ha M, Kim VN. Regulation of microRNA biogenesis. Nat Rev Mol Cell Biol. 2014;15(8):509–24. 10.1038/nrm3838 25027649

[2] Kozomara A, Griffiths-Jones S. miRBase: annotating high confidence microRNAs using deep sequencing data. Nucleic Acids Res. 2014;42(Database issue):D68–73. 10.1093/nar/gkt1181 24275495 PMC3965103

[3] Meister G. Argonaute proteins: functional insights and emerging roles. Nat Rev Genet. 2013;14(7):447–59. 10.1038/nrg3462 23732335

[4] Kincaid RP, Sullivan CS. Virus-encoded microRNAs: an overview and a look to the future. PLoS Pathog. 2012;8(12):e1003018 10.1371/journal.ppat.1003018 23308061 PMC3534370

[5] Grundhoff A, Sullivan CS. Virus-encoded microRNAs. Virology. 2011;411(2):325–43. 10.1016/j.virol.2011.01.002 21277611 PMC3052296

[6] Skalsky RL, Cullen BR. Viruses, microRNAs, and host interactions. Annu Rev Microbiol. 2010;64:123–41. 10.1146/annurev.micro.112408.134243 20477536 PMC3621958

[7] Babcock GJ, Decker LL, Volk M, Thorley-Lawson DA. EBV persistence in memory B cells in vivo. Immunity. 1998;9(3):395–404. 9768759 10.1016/s1074-7613(00)80622-6

[8] Young LS, Rickinson AB. Epstein-Barr virus: 40 years on. Nat Rev Cancer. 2004;4(10):757–68. 10.1038/nrc1452 15510157

[9] Berger C, Hug M, Gysin C, Molinari L, Frei M, Bossart W, et al Distribution patterns of beta- and gamma-herpesviruses within Waldeyer's ring organs. Journal of Medical Virology. 2007;79(8):1147–52. 10.1002/jmv.20899 17597487

[10] Nadal D, Blasius M, Niggli FK, Meier G, Berger C. Epstein-Barr virus (EBV) DNA levels in palatine tonsils and autologous serum from EBV carriers. Journal of Medical Virology. 2002;67(1):54–8. 11920818 10.1002/jmv.2192

[11] Delecluse HJ, Feederle R, O'Sullivan B, Taniere P. Epstein Barr virus-associated tumours: an update for the attention of the working pathologist. J Clin Pathol. 2007;60(12):1358–64. 10.1136/jcp.2006.044586 17873116 PMC2095566

[12] Hudnall SD, Ge Y, Wei L, Yang NP, Wang HQ, Chen T. Distribution and phenotype of Epstein-Barr virus-infected cells in human pharyngeal tonsils. Mod Pathol. 2005;18(4):519–27. 10.1038/modpathol.3800369 15696119

[13] Ikeda T, Kobayashi R, Horiuchi M, Nagata Y, Hasegawa M, Mizuno F, et al Detection of lymphocytes productively infected with Epstein-Barr virus in non-neoplastic tonsils. J Gen Virol. 2000;81(Pt 5):1211–6. 10.1099/0022-1317-81-5-1211 10769062

[14] Kang D, Skalsky RL, Cullen BR. EBV BART MicroRNAs Target Multiple Pro-apoptotic Cellular Genes to Promote Epithelial Cell Survival. PLoS Pathog. 2015;11(6):e1004979 10.1371/journal.ppat.1004979 26070070 PMC4466530

[15] Qiu J, Cosmopoulos K, Pegtel M, Hopmans E, Murray P, Middeldorp J, et al A novel persistence associated EBV miRNA expression profile is disrupted in neoplasia. PLoS Pathog. 2011;7(8):e1002193 10.1371/journal.ppat.1002193 21901094 PMC3161978

[16] Skalsky RL, Corcoran DL, Gottwein E, Frank CL, Kang D, Hafner M, et al The viral and cellular microRNA targetome in lymphoblastoid cell lines. PLoS Pathog. 2012;8(1):e1002484 10.1371/journal.ppat.1002484 22291592 PMC3266933

[17] Cai X, Schafer A, Lu S, Bilello JP, Desrosiers RC, Edwards R, et al Epstein-Barr virus microRNAs are evolutionarily conserved and differentially expressed. PLoS Pathog. 2006;2(3):e23 10.1371/journal.ppat.0020023 16557291 PMC1409806

[18] Hage E, Gerd Liebert U, Bergs S, Ganzenmueller T, Heim A. Human mastadenovirus type 70: a novel, multiple recombinant species D mastadenovirus isolated from diarrhoeal faeces of a haematopoietic stem cell transplantation recipient. J Gen Virol. 2015;96(9):2734–42. 10.1099/vir.0.000196 26002300

[19] Robinson CM, Singh G, Lee JY, Dehghan S, Rajaiya J, Liu EB, et al Molecular evolution of human adenoviruses. Sci Rep. 2013;3:1812 10.1038/srep01812 23657240 PMC3648800

[20] Adhikary AK, Banik U. Human adenovirus type 8: the major agent of epidemic keratoconjunctivitis (EKC). J Clin Virol. 2014;61(4):477–86. 10.1016/j.jcv.2014.10.015 25464969

[21] Guo L, Gonzalez R, Zhou H, Wu C, Vernet G, Wang Z, et al Detection of three human adenovirus species in adults with acute respiratory infection in China. Eur J Clin Microbiol Infect Dis. 2012;31(6):1051–8. 10.1007/s10096-011-1406-8 21964587 PMC7087767

[22] Lu L, Jia R, Zhong H, Xu M, Su L, Cao L, et al Molecular characterization and multiple infections of rotavirus, norovirus, sapovirus, astrovirus and adenovirus in outpatients with sporadic gastroenteritis in Shanghai, China, 2010–2011. Arch Virol. 2015;160(5):1229–38. 10.1007/s00705-015-2387-1 25772574

[23] Richman DD, Whitley RJ, Hayden FG. Clinical virology. 3rd ed Washington, DC: ASM Press; 2009 p. xvi, 1375.

[24] Alkhalaf MA, Guiver M, Cooper RJ. Prevalence and quantitation of adenovirus DNA from human tonsil and adenoid tissues. J Med Virol. 2013;85(11):1947–54. 10.1002/jmv.23678 23852770

[25] Assadian F, Sandstrom K, Bondeson K, Laurell G, Lidian A, Svensson C, et al Distribution and Molecular Characterization of Human Adenovirus and Epstein-Barr Virus Infections in Tonsillar Lymphocytes Isolated from Patients Diagnosed with Tonsillar Diseases. PLoS One. 2016;11(5):e0154814 10.1371/journal.pone.0154814 27136093 PMC4852932

[26] Garnett CT, Talekar G, Mahr JA, Huang W, Zhang Y, Ornelles DA, et al Latent species C adenoviruses in human tonsil tissues. J Virol. 2009;83(6):2417–28. 10.1128/JVI.02392-08 19109384 PMC2648257

[27] Proenca-Modena JL, Pereira Valera FC, Jacob MG, Buzatto GP, Saturno TH, Lopes L, et al High rates of detection of respiratory viruses in tonsillar tissues from children with chronic adenotonsillar disease. PLoS One. 2012;7(8):e42136 10.1371/journal.pone.0042136 22870291 PMC3411673

[28] van der Veen J, Lambriex M. Relationship of adenovirus to lymphocytes in naturally infected human tonsils and adenoids. Infect Immun. 1973;7(4):604–9. 4796933 10.1128/iai.7.4.604-609.1973 PMC422731

[29] Garnett CT, Erdman D, Xu W, Gooding LR. Prevalence and quantitation of species C adenovirus DNA in human mucosal lymphocytes. J Virol. 2002;76(21):10608–16. 10.1128/JVI.76.21.10608-10616.2002 12368303 PMC136639

[30] Iwakiri D. Multifunctional non-coding Epstein-Barr virus encoded RNAs (EBERs) contribute to viral pathogenesis. Virus Res. 2016;212:30–8. 10.1016/j.virusres.2015.08.007 26292159

[31] Punga T, Kamel W, Akusjarvi G. Old and new functions for the adenovirus virus-associated RNAs. Future Virology. 2013;8(4):343–56.

[32] Rosa MD, Gottlieb E, Lerner MR, Steitz JA. Striking similarities are exhibited by two small Epstein-Barr virus-encoded ribonucleic acids and the adenovirus-associated ribonucleic acids VAI and VAII. Mol Cell Biol. 1981;1(9):785–96. 9279391 10.1128/mcb.1.9.785-796.1981 PMC369362

[33] Tycowski KT, Guo YE, Lee N, Moss WN, Vallery TK, Xie MY, et al Viral noncoding RNAs: more surprises. Genes &Development. 2015;29(6):567–84. 25792595 10.1101/gad.259077.115 PMC4378190

[34] Clarke PA, Schwemmle M, Schickinger J, Hilse K, Clemens MJ. Binding of Epstein-Barr virus small RNA EBER-1 to the double-stranded RNA-activated protein kinase DAI. Nucleic Acids Res. 1991;19(2):243–8. 1673026 10.1093/nar/19.2.243 PMC333586

[35] Sharp TV, Schwemmle M, Jeffrey I, Laing K, Mellor H, Proud CG, et al Comparative analysis of the regulation of the interferon-inducible protein kinase PKR by Epstein-Barr virus RNAs EBER-1 and EBER-2 and adenovirus VAI RNA. Nucleic Acids Res. 1993;21(19):4483–90. 7901835 10.1093/nar/21.19.4483 PMC311179

[36] Iwakiri D, Zhou L, Samanta M, Matsumoto M, Ebihara T, Seya T, et al Epstein-Barr virus (EBV)-encoded small RNA is released from EBV-infected cells and activates signaling from Toll-like receptor 3. J Exp Med. 2009;206(10):2091–9. 10.1084/jem.20081761 19720839 PMC2757889

[37] Nanbo A, Inoue K, Adachi-Takasawa K, Takada K. Epstein-Barr virus RNA confers resistance to interferon-alpha-induced apoptosis in Burkitt's lymphoma. EMBO J. 2002;21(5):954–65. 10.1093/emboj/21.5.954 11867523 PMC125896

[38] Samanta M, Iwakiri D, Takada K. Epstein-Barr virus-encoded small RNA induces IL-10 through RIG-I-mediated IRF-3 signaling. Oncogene. 2008;27(30):4150–60. 10.1038/onc.2008.75 18362887

[39] Pimienta G, Fok V, Haslip M, Nagy M, Takyar S, Steitz JA. Proteomics and Transcriptomics of BJAB Cells Expressing the Epstein-Barr Virus Noncoding RNAs EBER1 and EBER2. PLoS One. 2015;10(6):e0124638 10.1371/journal.pone.0124638 26121143 PMC4487896

[40] Andersson MG, Haasnoot PC, Xu N, Berenjian S, Berkhout B, Akusjarvi G. Suppression of RNA interference by adenovirus virus-associated RNA. J Virol. 2005;79(15):9556–65. 10.1128/JVI.79.15.9556-9565.2005 16014917 PMC1181602

[41] Sano M, Kato Y, Taira K. Sequence-specific interference by small RNAs derived from adenovirus VAI RNA. FEBS Lett. 2006;580(6):1553–64. 10.1016/j.febslet.2006.01.085 16472808

[42] Xu N, Segerman B, Zhou X, Akusjarvi G. Adenovirus virus-associated RNAII-derived small RNAs are efficiently incorporated into the rna-induced silencing complex and associate with polyribosomes. J Virol. 2007;81(19):10540–9. 10.1128/JVI.00885-07 17652395 PMC2045446

[43] Kamel W, Segerman B, Punga T, Akusjarvi G. Small RNA sequence analysis of adenovirus VA RNA-derived miRNAs reveals an unexpected serotype-specific difference in structure and abundance. PLoS One. 2014;9(8):e105746 10.1371/journal.pone.0105746 25144466 PMC4140831

[44] Kamel W, Segerman B, Oberg D, Punga T, Akusjarvi G. The adenovirus VA RNA-derived miRNAs are not essential for lytic virus growth in tissue culture cells. Nucleic Acids Res. 2013;41(9):4802–12. 10.1093/nar/gkt172 23525465 PMC3643585

[45] Alles J, Hasler D, Kazmi MA, Tesson M, Hamilton A, Schlegel L, et al Epstein-Barr Virus EBER Transcripts Affect miRNA-Mediated Regulation of Specific Targets and Are Processed to Small RNA Species. Non-Coding RNA. 2015;1:170–91. 29861423 10.3390/ncrna1030170 PMC5932547

[46] Hutzinger R, Feederle R, Mrazek J, Schiefermeier N, Balwierz PJ, Zavolan M, et al Expression and processing of a small nucleolar RNA from the Epstein-Barr virus genome. PLoS Pathog. 2009;5(8):e1000547 10.1371/journal.ppat.1000547 19680535 PMC2718842

[47] Lung RW, Tong JH, To KF. Emerging roles of small Epstein-Barr virus derived non-coding RNAs in epithelial malignancy. Int J Mol Sci. 2013;14(9):17378–409. 10.3390/ijms140917378 23979421 PMC3794732

[48] Assadian F, Sandström K, Laurell G, Akusjärvi G, Punga T. Efficient isolation protocol for B and T lymphocytes from human palatine tonsils. J Vis Exp. 2015;(105). 10.3791/53374 PMC4692726 26650582

[49] Langmead B, Trapnell C, Pop M, Salzberg SL. Ultrafast and memory-efficient alignment of short DNA sequences to the human genome. Genome Biol. 2009;10(3):R25 10.1186/gb-2009-10-3-r25 19261174 PMC2690996

[50] Robinson JT, Thorvaldsdottir H, Winckler W, Guttman M, Lander ES, Getz G, et al Integrative genomics viewer. Nat Biotechnol. 2011;29(1):24–6. 10.1038/nbt.1754 21221095 PMC3346182

[51] Liao Y, Smyth GK, Shi W. featureCounts: an efficient general purpose program for assigning sequence reads to genomic features. Bioinformatics. 2014;30(7):923–30. 10.1093/bioinformatics/btt656 24227677

[52] Robinson MD, Oshlack A. A scaling normalization method for differential expression analysis of RNA-seq data. Genome Biol. 2010;11(3):R25 10.1186/gb-2010-11-3-r25 20196867 PMC2864565

[53] Jolliffe IT. Principal component analysis. 2nd ed New York: Springer; 2002 xxix, 487 p. p.

[54] Xing L, Kieff E. Epstein-Barr virus BHRF1 micro- and stable RNAs during latency III and after induction of replication. J Virol. 2007;81(18):9967–75. 10.1128/JVI.02244-06 17626073 PMC2045418

[55] Chen SJ, Chen GH, Chen YH, Liu CY, Chang KP, Chang YS, et al Characterization of Epstein-Barr virus miRNAome in nasopharyngeal carcinoma by deep sequencing. PLoS One. 2010;5(9). 10.1371/journal.pone.0012745 PMC2942828 20862214

[56] Hooykaas MJ, Kruse E, Wiertz EJ, Lebbink RJ. Comprehensive profiling of functional Epstein-Barr virus miRNA expression in human cell lines. BMC Genomics. 2016;17:644 10.1186/s12864-016-2978-6 27531524 PMC4987988

[57] Morin RD, O'Connor MD, Griffith M, Kuchenbauer F, Delaney A, Prabhu AL, et al Application of massively parallel sequencing to microRNA profiling and discovery in human embryonic stem cells. Genome Res. 2008;18(4):610–21. 10.1101/gr.7179508 18285502 PMC2279248

[58] Bellutti F, Kauer M, Kneidinger D, Lion T, Klein R. Identification of RISC-associated adenoviral microRNAs, a subset of their direct targets, and global changes in the targetome upon lytic adenovirus 5 infection. J Virol. 2015;89(3):1608–27. 10.1128/JVI.02336-14 25410853 PMC4300742

[59] Zhao H, Chen M, Tellgren-Roth C, Pettersson U. Fluctuating expression of microRNAs in adenovirus infected cells. Virology. 2015;478:99–111. 10.1016/j.virol.2015.01.033 25744056

[60] Furuse Y, Ornelles DA, Cullen BR. Persistently adenovirus-infected lymphoid cells express microRNAs derived from the viral VAI and especially VAII RNA. Virology. 2013;447(1–2):140–5. 10.1016/j.virol.2013.08.024 24210108 PMC3825519

[61] Vennstrom B, Pettersson U, Philipson L. Two initiation sites for adenovirus 5.5S RNA. Nucleic Acids Res. 1978;5(1):195–204. 643608 10.1093/nar/5.1.195 PMC341971

[62] Xu N, Gkountela S, Saeed K, Akusjarvi G. The 5'-end heterogeneity of adenovirus virus-associated RNAI contributes to the asymmetric guide strand incorporation into the RNA-induced silencing complex. Nucleic Acids Res. 2009;37(20):6950–9. 10.1093/nar/gkp764 19755500 PMC2777419

[63] Riley KJ, Rabinowitz GS, Steitz JA. Comprehensive analysis of Rhesus lymphocryptovirus microRNA expression. J Virol. 2010;84(10):5148–57. 10.1128/JVI.00110-10 20219930 PMC2863793

[64] Skalsky RL, Kang D, Linnstaedt SD, Cullen BR. Evolutionary conservation of primate lymphocryptovirus microRNA targets. J Virol. 2014;88(3):1617–35. 10.1128/JVI.02071-13 24257599 PMC3911612

[65] Umbach JL, Cullen BR. In-depth analysis of Kaposi's sarcoma-associated herpesvirus microRNA expression provides insights into the mammalian microRNA-processing machinery. J Virol. 2010;84(2):695–703. 10.1128/JVI.02013-09 19889781 PMC2798371

[66] Wu YQ, Chen DJ, He HB, Chen DS, Chen LL, Chen HC, et al Pseudorabies virus infected porcine epithelial cell line generates a diverse set of host microRNAs and a special cluster of viral microRNAs. PLoS One. 2012;7(1):e30988 10.1371/journal.pone.0030988 22292087 PMC3264653

[67] Lee LW, Zhang S, Etheridge A, Ma L, Martin D, Galas D, et al Complexity of the microRNA repertoire revealed by next-generation sequencing. Rna. 2010;16(11):2170–80. 10.1261/rna.2225110 20876832 PMC2957056

[68] Gu S, Jin L, Zhang Y, Huang Y, Zhang F, Valdmanis PN, et al The loop position of shRNAs and pre-miRNAs is critical for the accuracy of dicer processing in vivo. Cell. 2012;151(4):900–11. 10.1016/j.cell.2012.09.042 23141545 PMC3499986

[69] Li Z, Chen X, Li L, Liu S, Yang L, Ma X, et al EBV encoded miR-BHRF1-1 potentiates viral lytic replication by downregulating host p53 in nasopharyngeal carcinoma. Int J Biochem Cell Biol. 2012;44(2):275–9. 10.1016/j.biocel.2011.11.007 22108199

[70] Barth S, Pfuhl T, Mamiani A, Ehses C, Roemer K, Kremmer E, et al Epstein-Barr virus-encoded microRNA miR-BART2 down-regulates the viral DNA polymerase BALF5. Nucleic Acids Res. 2008;36(2):666–75. 10.1093/nar/gkm1080 18073197 PMC2241876

[71] Lo AK, To KF, Lo KW, Lung RW, Hui JW, Liao G, et al Modulation of LMP1 protein expression by EBV-encoded microRNAs. Proc Natl Acad Sci U S A. 2007;104(41):16164–9. 10.1073/pnas.0702896104 17911266 PMC2042179

[72] Pfeffer S, Sewer A, Lagos-Quintana M, Sheridan R, Sander C, Grasser FA, et al Identification of microRNAs of the herpesvirus family. Nat Methods. 2005;2(4):269–76. 10.1038/nmeth746 15782219

[73] Ambrosio MR, Navari M, Di Lisio L, Leon EA, Onnis A, Gazaneo S, et al The Epstein Barr-encoded BART-6-3p microRNA affects regulation of cell growth and immuno response in Burkitt lymphoma. Infect Agent Cancer. 2014;9:12 10.1186/1750-9378-9-12 24731550 PMC4005456

[74] Bavi P, Uddin S, Bu R, Ahmed M, Abubaker J, Balde V, et al The biological and clinical impact of inhibition of NF-kappaB-initiated apoptosis in diffuse large B cell lymphoma (DLBCL). J Pathol. 2011;224(3):355–66. 10.1002/path.2864 21506127

[75] Dirmeier U, Hoffmann R, Kilger E, Schultheiss U, Briseno C, Gires O, et al Latent membrane protein 1 of Epstein-Barr virus coordinately regulates proliferation with control of apoptosis. Oncogene. 2005;24(10):1711–7. 10.1038/sj.onc.1208367 15674340

[76] Jun Lu, Bidisha Chanda and Ai Kotani (2012). Epstein-Barr Virus-Encoded miRNAs in Epstein-Barr Virus-Related Malignancy, Hematology—Science and Practice, Dr. Charles Lawrie (Ed.), InTech, http://www.intechopen.com/books/hematology-science-and-practice/ebv-encoded-mirnas-in-ebv-related-malignancy.

[77] Eliopoulos AG, Young LS. LMP1 structure and signal transduction. Semin Cancer Biol. 2001;11(6):435–44. 10.1006/scbi.2001.0410 11669605

[78] Roberts ML, Cooper NR. Activation of a ras-MAPK-dependent pathway by Epstein-Barr virus latent membrane protein 1 is essential for cellular transformation. Virology. 1998;240(1):93–9. 10.1006/viro.1997.8901 9448693

[79] Wong AM, Kong KL, Tsang JW, Kwong DL, Guan XY. Profiling of Epstein-Barr virus-encoded microRNAs in nasopharyngeal carcinoma reveals potential biomarkers and oncomirs. Cancer. 2012;118(3):698–710. 10.1002/cncr.26309 21720996

[80] Miller JR, Hocking AM, Brown JD, Moon RT. Mechanism and function of signal transduction by the Wnt/beta-catenin and Wnt/Ca2+ pathways. Oncogene. 1999;18(55):7860–72. 10.1038/sj.onc.1203245 10630639

[81] Nachmani D, Stern-Ginossar N, Sarid R, Mandelboim O. Diverse herpesvirus microRNAs target the stress-induced immune ligand MICB to escape recognition by natural killer cells. Cell Host Microbe. 2009;5(4):376–85. 10.1016/j.chom.2009.03.003 19380116

[82] Stern-Ginossar N, Elefant N, Zimmermann A, Wolf DG, Saleh N, Biton M, et al Host immune system gene targeting by a viral miRNA. Science. 2007;317(5836):376–81. 10.1126/science.1140956 17641203 PMC4283197

[83] Choy EY, Siu KL, Kok KH, Lung RW, Tsang CM, To KF, et al An Epstein-Barr virus-encoded microRNA targets PUMA to promote host cell survival. J Exp Med. 2008;205(11):2551–60. 10.1084/jem.20072581 18838543 PMC2571930

[84] Xia T, O'Hara A, Araujo I, Barreto J, Carvalho E, Sapucaia JB, et al EBV microRNAs in primary lymphomas and targeting of CXCL-11 by ebv-mir-BHRF1-3. Cancer Res. 2008;68(5):1436–42. 10.1158/0008-5472.CAN-07-5126 18316607 PMC2855641

[85] Ma Y, Mathews MB. Structure, function, and evolution of adenovirus-associated RNA: a phylogenetic approach. J Virol. 1996;70(8):5083–99. 8764016 10.1128/jvi.70.8.5083-5099.1996 PMC190463

