# Supplementary material for: Expression profile of Epstein-Barr virus and human adenovirus small RNAs in tonsillar B and T lymphocytes
Source: PLoS One. 2017 May 25;12(5):e0177275. doi: 10.1371/journal.pone.0177275 (PMC5444648; doi:10.1371/journal.pone.0177275)
Supplement: S1 Fig — The pie charts display the distribution of the mapped reads from EBV+ (A) and HAdV+ (B) samples. (PDF) [file pone.0177275.s001.pdf]

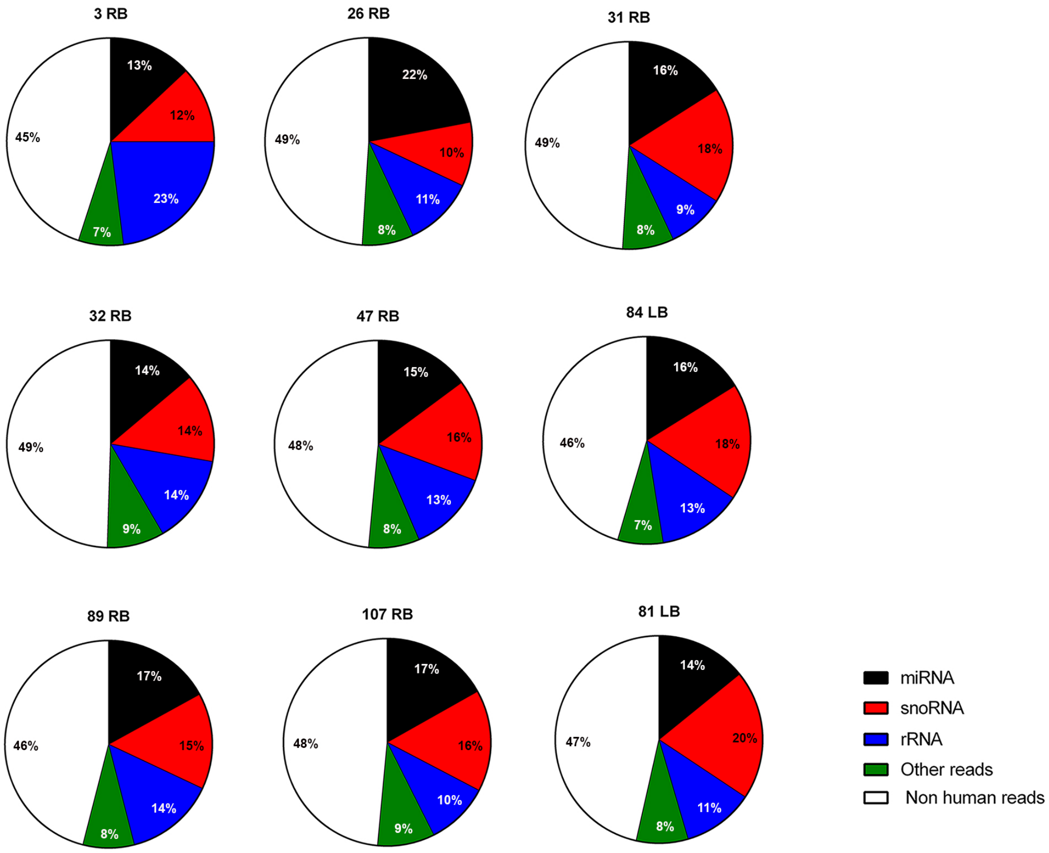

Figure S1A. Genotyping of the EBV+ sequencing reads aligned to human genome. The pie charts display the distribution of mapped reads from EBV+ patient samples.

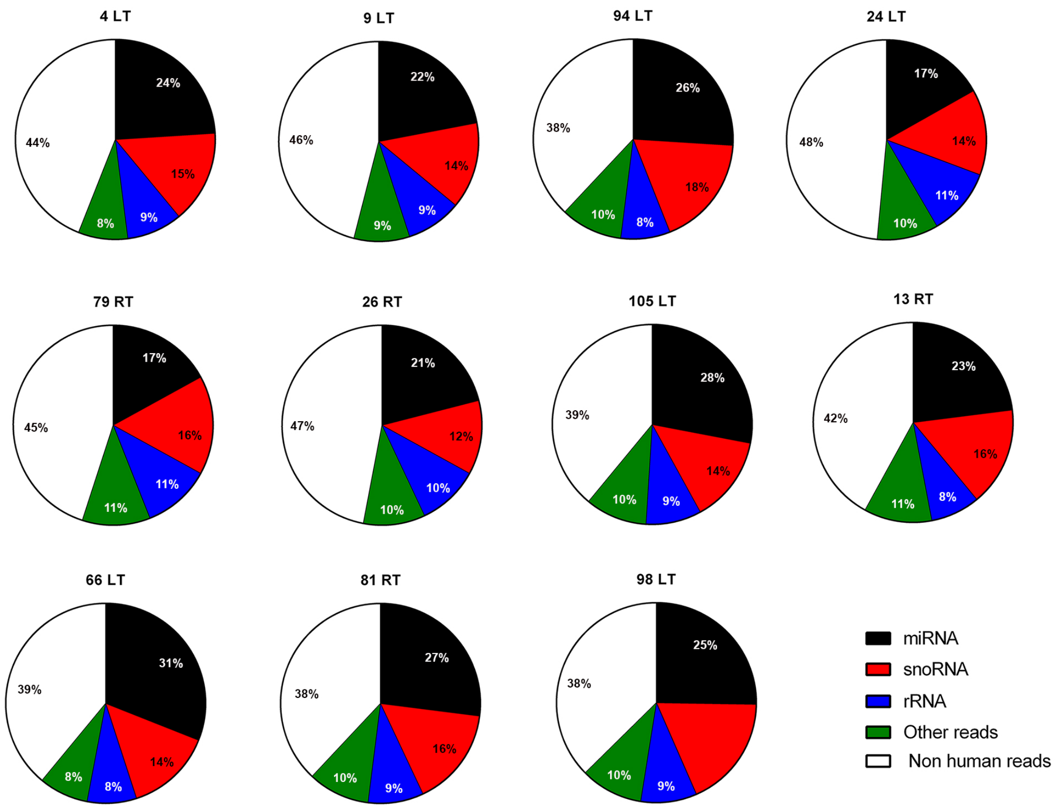

Figure S1B. Genotyping of the HAdV sequencing reads aligned to human genome. The pie charts display the distribution of the mapped reads from HAdV+ patient samples.
