# Supplementary material for: Expression profile of Epstein-Barr virus and human adenovirus small RNAs in tonsillar B and T lymphocytes
Source: PLoS One. 2017 May 25;12(5):e0177275. doi: 10.1371/journal.pone.0177275 (PMC5444648; doi:10.1371/journal.pone.0177275)
Supplement: S2 Fig — The PCA analysis was performed on the data set normalized based on the TMM method. For the T cell patient samples the diagnosis (tonsillar hypertrophy versus chronic/recurrent tonsillitis) are shown circled. (PDF) [file pone.0177275.s002.pdf]

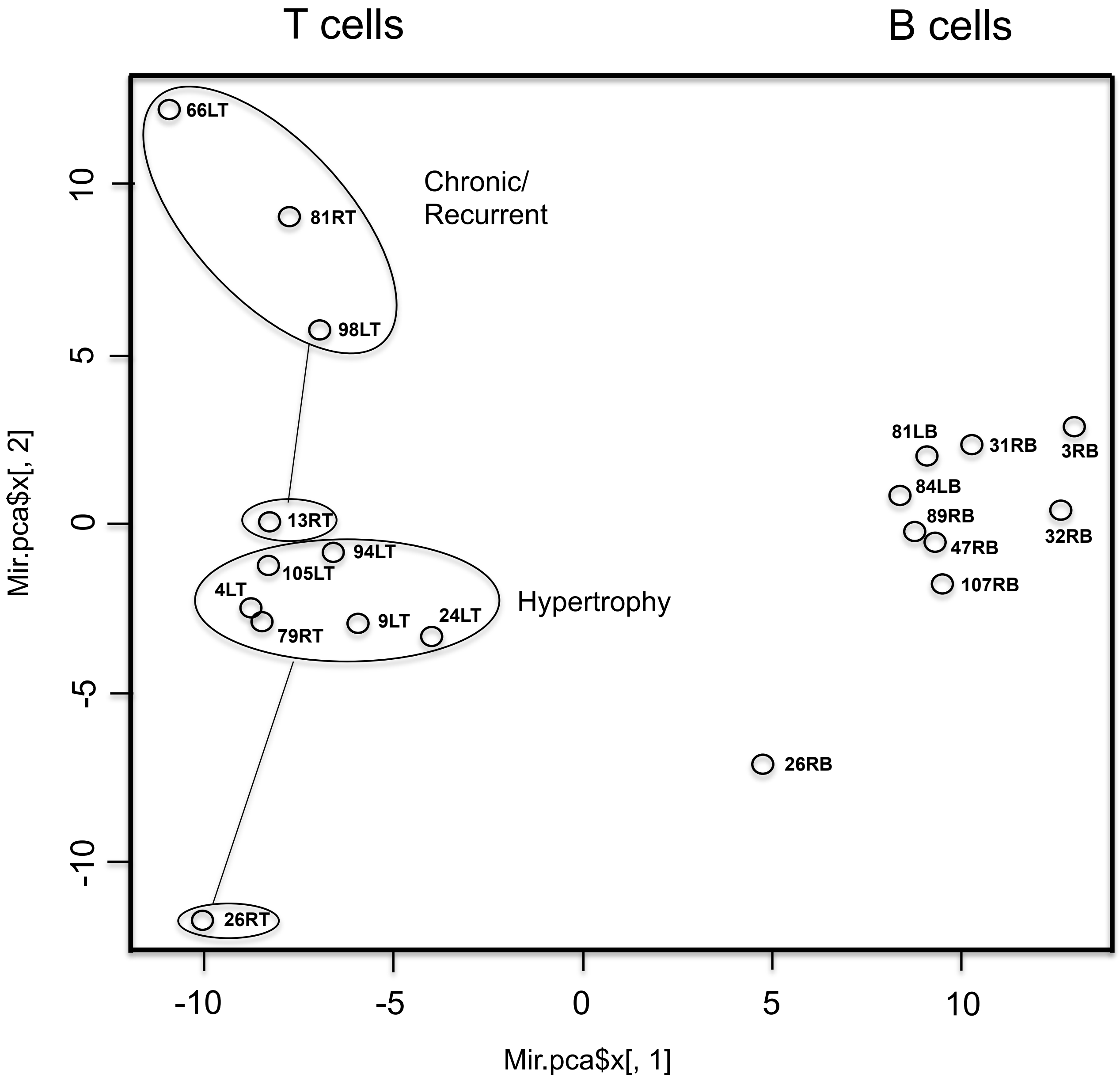

Figure S2. Principle component analysis (PCA) of cellular miRNA expression in the tonsillar B and T lymphocytes. The PCA analysis was performed on the data set normalized based on the TMM method. For the T cell patient samples the Diagnosis (tonsillar hypertrophy versus chronic/recurrent tonsillitis) are shown circled.
