# Supplementary material for: Expression profile of Epstein-Barr virus and human adenovirus small RNAs in tonsillar B and T lymphocytes
Source: PLoS One. 2017 May 25;12(5):e0177275. doi: 10.1371/journal.pone.0177275 (PMC5444648; doi:10.1371/journal.pone.0177275)
Supplement: S3 Fig — The annotated BART16-5p miRNA is shown in red whereas the new BART16-3p and BART16b-5p are shown in blue. (PDF) [file pone.0177275.s003.pdf]

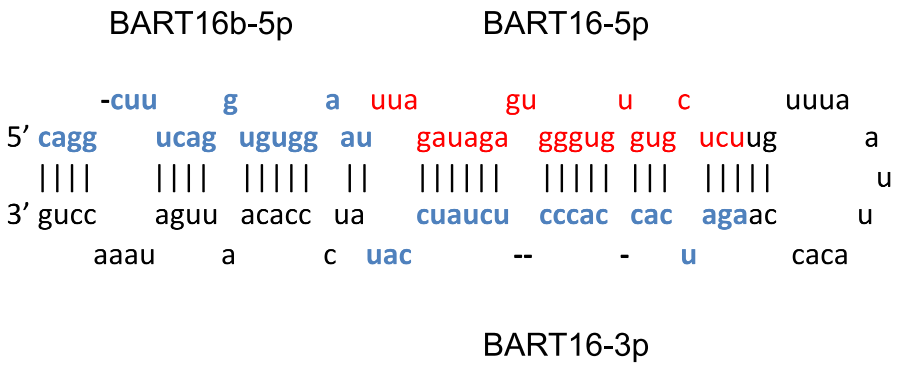

Figure S3. The proposed EBV miR-BART16 precursor RNA. The annotated BART16-5p miRNA is shown in red whereas the new BART16-3p and BART16b-5p are shown in blue.
