# Supplementary material for: Expression profile of Epstein-Barr virus and human adenovirus small RNAs in tonsillar B and T lymphocytes
Source: PLoS One. 2017 May 25;12(5):e0177275. doi: 10.1371/journal.pone.0177275 (PMC5444648; doi:10.1371/journal.pone.0177275)
Supplement: S4 Fig — The boxes indicate the relative distribution of reads mapped to 5´or 3´regions of EBER1 and EBER2. (PDF) [file pone.0177275.s004.pdf]

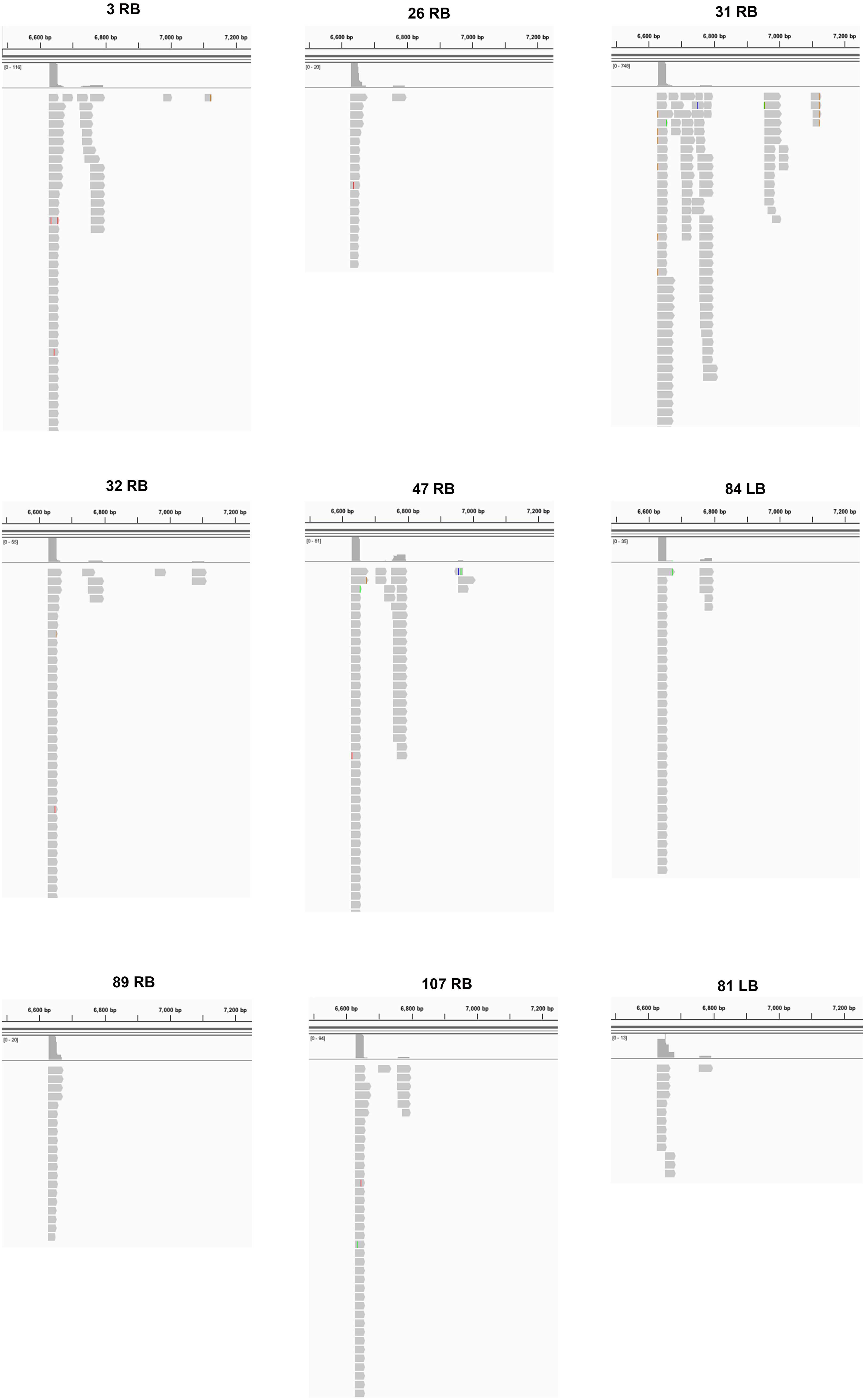

Figure S4. Coverage of EBER derived small RNA reads in the EBV+ B lymphocytes from the different patients. The boxes indicate the relative distribution of reads mapped to 5´or 3´regions of EBER1 and EBER2.
