# Supplementary material for: Expression profile of Epstein-Barr virus and human adenovirus small RNAs in tonsillar B and T lymphocytes
Source: PLoS One. 2017 May 25;12(5):e0177275. doi: 10.1371/journal.pone.0177275 (PMC5444648; doi:10.1371/journal.pone.0177275)
Supplement: S1 Table — The asterisk denotes new miRNAs not annotated in miRBase v21. The highest-expressed miRNAs in each sample are shown in bold. (PDF) [file pone.0177275.s005.pdf]

**EBV miRNA**	**Start**	**End**	**% of relative expression of EBV miRNAs in the individual samples**								
			**3RB**	**26RB**	**31RB**	**32RB**	**47RB**	**84LB**	**89RB**	**107 RB**	**81 LB**
**BHRF1-1-5p**	41474	41495		0.26	0.01						
**BHRF1-1-3p***	41512	41534			0.01						
**BHRF1-2-5p**	42853	42874									
**BHRF1-2-3p**	42888	42909									
**BHRF1-3**	42968	42989									
**BART3-5p**	139087	139107	0.03	0.13	0.10	0.08				0.36	
**BART3-3p**	139124	139145	0.18	0.39	0.21		0.25	0.42	0.46		
**BART4-5p**	139228	139249	0.37	0.26	0.47		0.25	0.84	0.23	0.72	
**BART4-3p**	139266	139288		0.13		0.08					
**BART1-5p**	139351	139374	1.34	1.57	1.02	1.28	0.76	2.95	0.93	0.97	0.41
**BART1-3p**	139387	139408	0.42	1.57	0.43	0.48	0.38		0.46	0.72	
**BART15-5p***	139519	139542		0.39	0.07	0.24		1.27			
**BART15-3p**	139553	139574	0.03		0.03	0.00			0.46		0.41
**BART5-5p**	139675	139698	0.39	0.65	0.37	0.16	0.25	0.42		0.30	0.41
**BART5-3p**	139717	139734	0.00		0.01						
**BART16b-5p***	139775	139794	0.16		0.00						
**BART16-5p**	139795	139818	1.89	1.31	1.96	1.20	2.40	4.22	0.93	2.17	0.41
**BART16-3p***	139834	139850	0.03		0.01						
**BART17-5p**	139915	139936	**18.45**	**11.10**	**17.49**	**15.17**	**10.61**	**8.86**	**13.46**	**15.28**	**13.93**
**BART17-3p**	139953	139975	0.71	0.91	0.76	0.24	0.51		1.39	0.97	0.41
**BART6-5p**	140033	140054	0.45	0.26	0.35	0.24	0.51		0.46	0.42	0.41
**BART6-3p**	140072	140093	**22.45**	**21.02**	**26.59**	**27.93**	**30.68**	**18.14**	**25.06**	**28.32**	**31.97**
**BART21-5p**	145514	145534			0.01						
**BART21-3p**	145548	145569	0.05		0.02		0.13	0.42			
**BART18-5p**	145962	145983	0.05	0.13	0.06	0.24	0.13			0.12	

**BART18-3p**	145998	146019	0.11	0.39	0.30	0.48		0.42	0.23	0.42	
**BART7-5p**	146439	146460	**3.58**	**4.18**	**3.17**	**2.65**	**1.77**	**5.91**	**4.41**	**2.60**	**4.51**
**BART7-3p**	146475	146496	1.66	1.04	1.03	1.36	3.54	2.95	1.86	1.09	0.41
**BART8-5p**	146772	146793	**4.97**	**5.35**	**3.39**	**6.66**	**5.30**	**7.59**	**8.58**	**3.74**	**6.56**
**BART8-3p**	146807	146829	2.47	2.61	1.94	2.17	3.91	4.64	3.71	3.74	2.87
**BART9-5p**	146959	146980	0.89	2.09	0.61	0.32	1.39	1.69	0.46	0.36	3.28
**BART9-3p**	146997	147019	0.58	0.13	0.68	0.40	0.13	0.84	0.23	1.15	
**BART22-5p***	147169	147190	0.18		0.15		0.25				
**BART22-3p**	147203	147225	3.26	4.83	2.33	2.81	1.52	2.11	4.64	2.66	3.28
**BART10-5p**	147321	147342		0.13	0.03						
**BART10-3p**	147356	147378	1.71	1.83	1.28	1.04	3.16	2.95	2.09	1.93	1.64
**BART11-5p**	147537	147560	1.37	2.48	1.15	1.20	2.78	3.80	0.23	0.72	1.23
**BART11-3p**	147575	147595	**5.15**	**6.79**	**7.50**	**8.27**	**7.45**	**10.97**	**6.96**	**7.55**	**10.25**
**BART12-5p***	147900	147922	0.24	0.52	0.44	0.40	0.38		0.46	0.12	0.41
**BART12-3p**	147936	147957	0.63	0.26	0.63	1.04	0.38	2.95	0.46	0.42	0.41
**BART19-5p**	148215	148237	**12.59**	**10.57**	**10.72**	**10.59**	**8.33**		**10.67**	**7.13**	**7.79**
**BART19-3p**	148254	148274	0.79	1.04	0.47	0.80	0.63	0.42	0.93	0.79	0.41
**BART20-5p**	148339	148359	0.08								
**BART20-3p**	148374	148395									
**BART13-5p**	148526	148547	**5.68**	**6.66**	**8.87**	**5.86**	**6.57**	**11.81**	**5.80**	**10.81**	**4.51**
**BART13-3p**	148563	148585		0.39	0.05	0.16			0.93	0.06	
**BART14-5p**	148744	148765	0.29	0.26	0.22	0.08				0.18	
**BART14-3p**	148778	148799	4.02	4.05	2.59	4.09	3.41	1.27	2.78	2.66	1.23
**BART2-5p**	152747	152768	2.58	3.92	2.35	2.01	2.15	2.11	0.70	1.39	2.46
**BART2-3p**	152783	152806	0.18	0.39	0.13	0.24	0.13			0.12	0.41

Table S1: The expression level of the miRNAs in the individual patient samples is shown as the percentage of each miRNA relative to the total miRNA content. The asterisk denotes new miRNAs not annotated in miRBase v21. The highest-expressed miRNAs in each sample are shown in bold.
