# Supplementary material for: Expression profile of Epstein-Barr virus and human adenovirus small RNAs in tonsillar B and T lymphocytes
Source: PLoS One. 2017 May 25;12(5):e0177275. doi: 10.1371/journal.pone.0177275 (PMC5444648; doi:10.1371/journal.pone.0177275)
Supplement: S2 Table — The expression level of the small RNAs/miRNAs in individual patients is shown as the percentage that each RNA contributes to the total EBV specific small RNA pool. (PDF) [file pone.0177275.s006.pdf]

EBV	Samples								
	**3RB**	**26RB**	**31RB**	**32RB**	**47RB**	**84LB**	**89RB**	**107RB**	**81LB**
**EBER1-derived small RNAs**	3.4	2.6	4.5	4.4	11.6	13.1	5.2	5.5	3.8
**EBER2-derived small RNAs**	0.0	0.0	0.1	0.1	0.2	0.0	0.0	0.0	0.0
**miR-BART17-5p**	17.4	10.4	16.2	14.3	9.3	7.1	12.4	14.3	11.7
**miR-BART6-3p**	21.2	19.8	24.7	26.4	27.0	14.5	23.2	26.5	26.9
**v-snoRNA1-derived small RNAs**	2.2	3.4	2.6	1.1	0.2	7.1	2.4	1.0	12.1

Table S2. Expression level of the EBER- and v-snoRNA1-derived small RNAs and the highest expressed BART miRNAs in the EBV-infected B lymphocyte patient samples. The expression level of the small RNAs/miRNAs in individual patients is shown as the percentage that each RNA contributes to the total EBVspecific small RNA pool.
